# Machine learning-enabled phenotyping for GWAS and TWAS of WUE traits in 869 field-grown sorghum accessions

**DOI:** 10.1093/plphys/kiab346

**Published:** 2021-07-27

**Authors:** John N Ferguson, Samuel B Fernandes, Brandon Monier, Nathan D Miller, Dylan Allen, Anna Dmitrieva, Peter Schmuker, Roberto Lozano, Ravi Valluru, Edward S Buckler, Michael A Gore, Patrick J Brown, Edgar P Spalding, Andrew D B Leakey

**Affiliations:** 1 Institute for Genomic Biology, University of Illinois at Urbana-Champaign, Urbana, Illinois 61901, USA; 2 Institute for Genomic Diversity, Cornell University, Ithaca, New York 14853, USA; 3 Department of Botany, University of Wisconsin, Madison, Wisconsin 53706, USA; 4 Plant Breeding and Genetics Section, School of Integrative Plant Science, Cornell University, Ithaca, New York 14853, USA; 5 Department of Crop Sciences, University of Illinois at Urbana-Champaign, Urbana, Illinois 61901, USA; 6 Department of Plant Biology, University of Illinois at Urbana-Champaign, Urbana, Illinois 61901, USA

## Abstract

Sorghum (*Sorghum bicolor*) is a model C_4_ crop made experimentally tractable by extensive genomic and genetic resources. Biomass sorghum is studied as a feedstock for biofuel and forage. Mechanistic modeling suggests that reducing stomatal conductance (*g_s_*) could improve sorghum intrinsic water use efficiency (iWUE) and biomass production. Phenotyping to discover genotype-to-phenotype associations remains a bottleneck in understanding the mechanistic basis for natural variation in *g_s_* and iWUE. This study addressed multiple methodological limitations. Optical tomography and a machine learning tool were combined to measure stomatal density (SD). This was combined with rapid measurements of leaf photosynthetic gas exchange and specific leaf area (SLA). These traits were the subject of genome-wide association study and transcriptome-wide association study across 869 field-grown biomass sorghum accessions. The ratio of intracellular to ambient CO_2_ was genetically correlated with SD, SLA, *g_s_*, and biomass production. Plasticity in SD and SLA was interrelated with each other and with productivity across wet and dry growing seasons. Moderate-to-high heritability of traits studied across the large mapping population validated associations between DNA sequence variation or RNA transcript abundance and trait variation. A total of 394 unique genes underpinning variation in WUE-related traits are described with higher confidence because they were identified in multiple independent tests. This list was enriched in genes whose Arabidopsis (*Arabidopsis thaliana*) putative orthologs have functions related to stomatal or leaf development and leaf gas exchange, as well as genes with nonsynonymous/missense variants. These advances in methodology and knowledge will facilitate improving C4 crop WUE.

## Teaser

Rapid phenotyping and machine learning-enabled GWAS and TWAS for 869 biomass sorghum accessions reveal relationships between water use efficiency traits and biomass in both wet and dry years.

## Introduction

Global climatic change is subjecting agricultural regions to elevated atmospheric vapor pressure deficits (Yuan et al., 2019) and patterns of precipitation that lead to greater or more frequent drought stress (IPCC, 2014, 2018). Water use efficiency (WUE; the ratio of carbon gain to water loss) is a key target trait for crop improvement to improve production and sustainable water use (Bailey-Serres et al., 2019; Leakey et al., 2019). C_4_ crops, including maize (*Zea mays*), sorghum (*Sorghum bicolor*), sugarcane (*Saccharum officinarum*), millet (*Panicum miliaceum*), and *Miscanthus*, are heavily studied as sources of food, feed, fuel, and fiber. However, less research has been directed toward understanding and improving WUE and its component traits in C_4_ crops, possibly because they already achieve high WUE as a result of the CO_2_ concentrating mechanism they possess (DeLucia et al., 2019). Nevertheless, mechanistic modeling suggests that enhancing intrinsic WUE (iWUE) by reducing stomatal conductance (*g_s_*) while maintaining rates of net CO_2_ assimilation (*A_N_*) can increase biomass production in C_4_ as well as C_3_ species across a broad range of environmental conditions (Truong et al., 2017; Leakey et al., 2019). These benefits will become greater as atmospheric [CO_2_] continues to rise. Compiling surveys of natural variation in C_4_ species, including grain sorghum (Kapanigowda et al., 2013), demonstrated that *g_s_* could explain substantially more variation in iWUE than *A_N_* (Leakey et al., 2019).

Sorghum is a model C_4_ crop made experimentally tractable by extensive genomic and genetic resources (Paterson et al., 2009;Morris et al., 2013). Biomass sorghum has considerable potential as a biofuel feedstock in addition to being grown for forage (Castro et al., 2015). This study aimed to address key knowledge gaps regarding natural variation in iWUE and related traits across diverse biomass sorghum accessions, including evaluation of heritability, environmental effects, trait correlations, and associations between DNA sequence variation or RNA transcript abundance and trait values. iWUE was studied alongside its component traits (*A_N_* and *g_s_*) plus stomatal density (SD) and specific leaf area (SLA) since these anatomical and allometric traits are known to influence leaf physiology.

Stomata open and close to regulate the rate of CO_2_ and water vapor exchange between leaves and the atmosphere (Cowan and Farquhar, 1977). These fluxes are also influenced by the size and density of stomata (Franks and Beerling, 2009). Empirical data show that SD is positively correlated with *g_s_* in a number of species (Anderson and Briske, 1990; Pearce et al., 2006). Molecular mechanisms controlling stomatal morphology and patterning have been elucidated in Arabidopsis (*Arabidopsis thaliana*; Chater et al., 2017). This has been combined with an understanding of how *g_s_* and WUE are linked to stomatal physiology to develop C_3_ plants with improved WUE. For example, the expression of species-specific putative orthologs of the *A. thaliana EPIDERMAL PATTERNING FACTOR 1* (*EPF1*) gene has been targeted to reduce *g_s_* through reduced SD, thereby improving WUE in barley (Hughes et al., 2017), rice (Caine et al., 2019; Mohammed et al., 2019), wheat (Dunn et al., 2019), and poplar (Wang et al., 2016a). The majority of cultivated crops are grasses (Leff et al., 2004). Stomatal morphology and development in grasses is markedly different from that of dicotyledonous species, for example, *A. thaliana*, and reflects specific selective pressures (Hetherington and Ian Woodward, 2003). Consequently, while in some instances, the molecular underpinnings of these traits are conserved between *A. thaliana* and grasses (Hughes et al., 2017; Caine et al., 2019; Dunn et al., 2019; Mohammed et al., 2019), emerging evidence suggests the biological functioning of key stomatal genes can be divergent between the lineages (Raissig et al., 2017; Abrash et al., 2018). As such, improving our understanding of grass-specific genes that regulate stomatal development and patterning will expedite efforts to improve WUE in crops. The need to address this knowledge gap is greatest in C_4_ species.

SLA is the ratio of leaf area to leaf mass, which combines information on leaf thickness and leaf density (John et al., 2017). It is a key trait in the leaf economic spectrum that influences many traits, including photosynthesis, respiration, leaf construction costs, leaf life span, canopy light interception, and growth rates (Wright et al., 2001). Despite its importance, and that it can be measured easily, efforts to understand the genetic architecture of the trait through quantitative trait loci mapping or genome-wide association study (GWAS) have been limited (El-Lithy et al., 2004; Trachsel et al., 2010). But, correlations between SLA and SD have been observed in response to varying water supply (Xu and Zhou, 2008) and across intraspecific variation associated with adaptation to aridity (Carlson et al., 2016). The genetic and environmental control of SLA and its relationship to SD in C_4_ species are especially poorly understood.

Efforts to discover the genetic basis of traits that influence the sustainability and resilience of crop productivity, including iWUE, are constrained by bottlenecks in both phenotyping and discovery of associations between trait variation and DNA sequence variation or gene expression . Automation, remote sensing, and machine learning are increasingly being used to accelerate the measurement and/or quantification of key ecophysiological traits (e.g. Atkinson et al., 2017; Feldman et al.,2018; Qiao et al., 2019). Optical tomography has been proposed as a method for imaging cell patterning on leaf surfaces that is much more rapid than traditional methods of epidermal peels or imprinting (Haus et al., 2015). Identifying and counting stomatal complexes on the epidermis is the most time-consuming aspect of screening SD. A number of machine learning tools have been proposed for counting stomata (e.g. Fetter et al., 2019; Sakoda et al., 2019). However, proof of concept is still required for the use of optical tomography and an automatic stomatal counting tool suitable for use across the phenotypic variation associated with diverse genotypes of a grass species.

GWAS and transcriptome-wide association study (TWAS) are the popular methods that can identify genomic regions or genes for which variation in DNA sequence or gene expression are associated with quantitative variation in a trait of interest (Tian et al., 2017; Hirsch et al., 2014). The challenges associated with phenotyping traits associated with iWUE in C_4_ crops mean that these methods have only been applied in a limited number of cases (Ortiz et al., 2017; Feldman et al., 2018). But, even when phenotypic data are readily available, association studies are often challenging because many traits are highly polygenic, where a large number of genes each exerts a weak effect on the trait (Zhu et al., 2008). Larger mapping population sizes can improve statistical power to counteract this problem. But, multiple testing at many single nucleotide polymorphisms across the genome also creates a risk of false-positive results. Validating the function of candidate genes via reverse genetics remains the gold standard but is extremely slow. Approaches that can increase the confidence and efficiency of identification of candidate genes from association studies are therefore important. One simple approach is to prioritize genes identified in multiple independent tests. GWAS can be supplemented by TWAS, which tests for associations between variation in transcript abundance and phenotypic variation. Most recently, proof-of-concept for applying Fisher’s combined test to integrate GWAS and TWAS was provided by demonstrating how it increased the efficiency with which known causal genes could be “re-discovered” for well-studied maize kernel traits (Kremling et al., 2019). However, the application of the method to address knowledge gaps for traits such as iWUE is untested.

In summary, to address knowledge gaps about the physiology and genetics of natural variation in iWUE in C_4_ grasses, this study evaluated a diverse population of 869 biomass sorghum accessions grown in replicated trials over two growing seasons. To achieve this goal, a set of tools were developed, tested, and integrated. To break the phenotyping bottleneck for SD, optical tomography was adapted and tested as an imaging technology, and a custom machine learning software platform was developed to automatically identify and count stomatal complexes. This was combined with a rapid method to measure leaf-level gas exchange and SLA. Trait correlations were evaluated, and genes putatively underlying genetic variation in iWUE and related traits were identified through GWAS, TWAS, and an ensemble association mapping approach. Genes identified with the greatest confidence were tested for the presence of nonsynonymous/missense variants.

## Results

### Growing season climate

A diversity panel of 869 photoperiod-sensitive sorghum accessions (Supplemental Figure S1; Supplemental Table S1) was grown at field locations within a 5-km radius in 2016 (*N* = 2; Fisher and Energy Farms) and 2017 (*N* = 2; Maxwell and Energy Farms). Mean daytime maximum temperature was similar between 2016 (28.9°C) and 2017 (28.8°C). But, compared to the average growing season rainfall of 396 mm (Gelaro et al., 2017), 2017 was dry (174 mm), and 2016 was wet (529 mm; Supplemental Figure S2).

### High-throughput phenotyping metrics

A high-throughput approach for measurement of photosynthetic gas exchange (*g_s_*, *A_N_*, iWUE, and the ratio of intracellular to atmospheric CO_2_ concentration [*c_i_*/*c_a_*]), along with tissue sampling for SLA and SD, was performed on ∼220 leaves per day, allowing two leaves per replicate plot of every genotype in the population to be sampled through 9–10 d of work for each replicate field in a given year.

**Figure 1:**
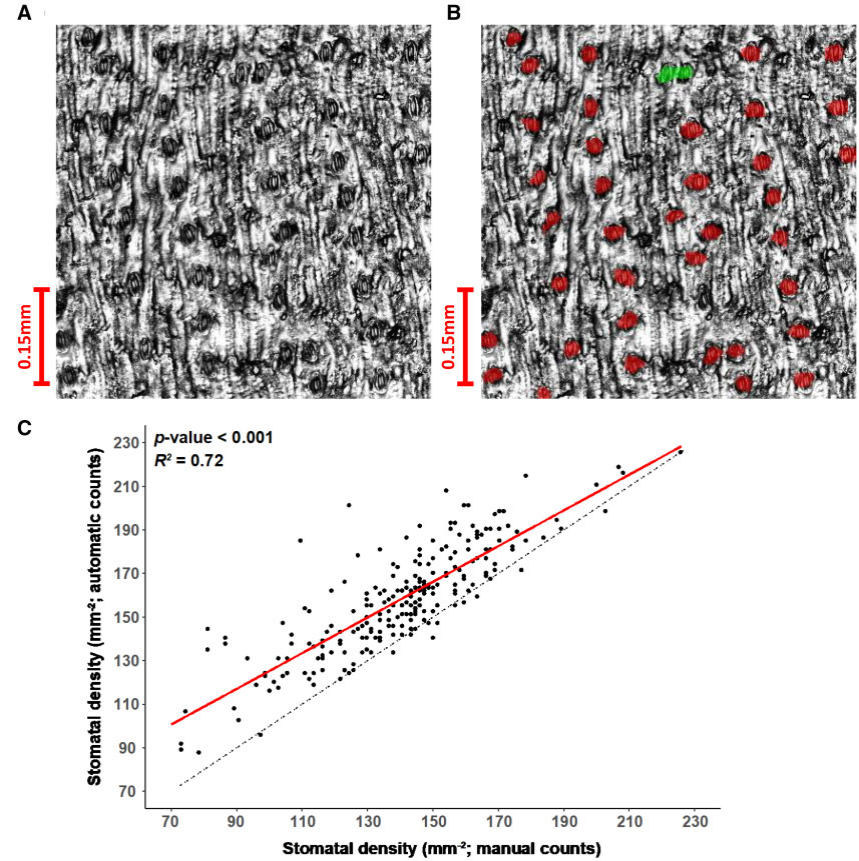
Demonstration of stomatal counting algorithm. A, Reflective intensity layer of an OT measurement of the abaxial epidermis of a sorghum leaf section. B, OT measurement from (A) overlaid with automatic detection of stomata (red). Automatically detected stomata in close proximity are highlighted in green. C, Association between the median SD of samples where stomates have been both manually and automatically counted. Four to six FOV were used to calculate the median values for each genotype by manual and automatic methods. A linear model regressing automatic counts on manual counts is fitted (red) and the standard error of the model is shown (gray). The associated *P-*value significance threshold and *r^2^* value of the model are inset.

Optical topometry (OT) was used to rapidly image 4–6 fields of view (FOV) from the abaxial surface of 4,169 leaves in 2016, and 3,211 leaves in 2017 without the need for sample preparation beyond adhesion to microscope slides with double-sided tape (∼250 FOVs per day per OT microscope; Figure 1A). High-throughput computing resources allowed SD to be assessed for each of the 33,355 FOVs in <24 h using a convolutional neural network that was trained to identify stomatal complexes in a rotationally invariant manner (Figure 1B;Supplemental Figure S3). In contrast, based on recent experience, manual counting of this image set would take an estimated 80 person-days. The median SD per leaf generated by this machine-vision platform was significantly positively correlated (*R*^2^ = 0.72, *P* < 0.001) with the median SD per leaf from human counting of 228 randomly selected ground truth samples (Figure 1C). Still, there was a bias toward the overestimation of SD by the computer because it occasionally mislabeled cells as stomatal complexes when they were actually pavement cells, especially on leaves with lower SD.

### Natural variation of WUE associated traits

**Figure 2:**
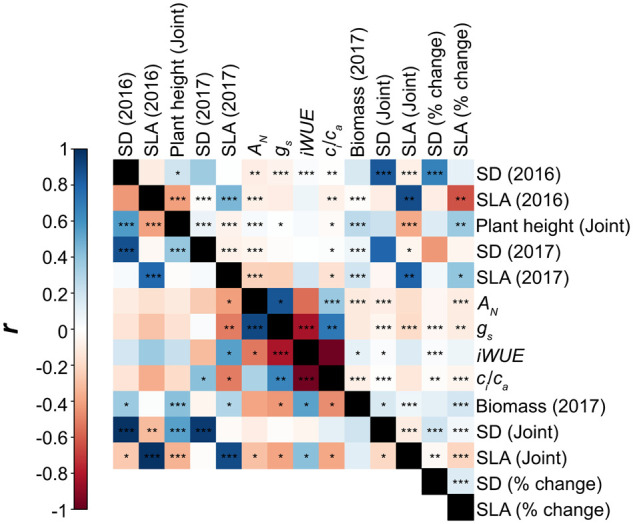
Correlogram demonstrating phenotypic (Pearson correlation; above diagonal) and genetic (Bivariate model; below diagonal) correlations for all measured parameters. Where appropriate, the associated model from which predicted means were extracted is indicated in parenthesis. The color of each square describes the correlation coefficient of each pairwise interaction. Significant correlations are denoted at the level of 0.001 (***), 0.01 (**), and 0.05 (*). Genetic and phenotypic correlations between traits extracted from each environment model are listed in Supplemental Table S2.

**Figure 3:**
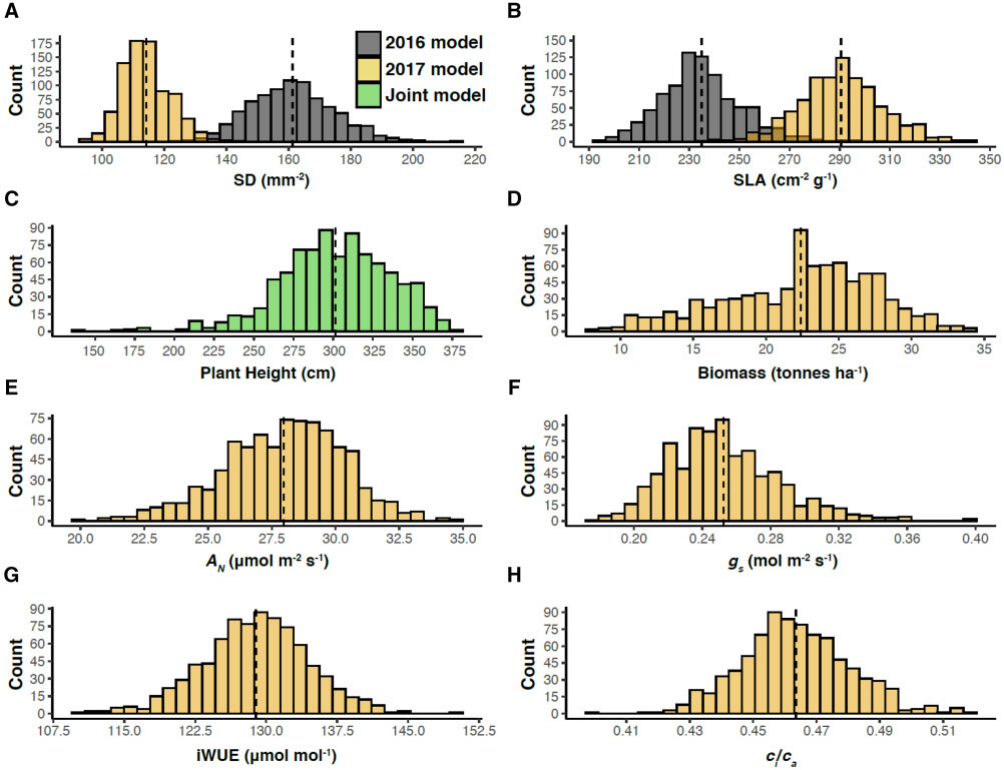
Histograms of variation in adjusted means of various traits. A, SD, (B) SLA, (C) plant height, (D) aboveground biomass (*A_N_*), (E) net photosynthesis (*A_N_*), (F) *g_s_*, (G) iWUE, and (H) *c_i_*/*c_a_*. The dashed vertical lines denote the population mean.

Data from 2017 allowed correlation analysis across the complete set of anatomical, physiological, and agronomic traits investigated (Figure 2). On a relative basis, the observed trait variation across the sorghum accessions was greatest for end-of-season biomass, followed by height, *g_s_*, *A_N_*, SD, SLA, iWUE, and *c_i_*/*c_a_* (Figure 3). *c_i_*/*c_a_* was genetically correlated with SD (*r_g_* = 0.41), SLA (*r_g_* = −0.52), *g_s_* (*r_g_* = 0.65), and biomass (*r_g_* = −0.47; Figure 2; Supplemental Table S2). When environmental variation was also accounted for in phenotypic correlations, *c_i_*/*c_a_* was correlated with SLA (*r*_p_ = −0.15), *g_s_* (*r_p_* = 0.72), *A_N_* (*r_p_* = 0.38), and biomass (*r_p_* = −0.08), although to a weaker degree (Figure 2;Supplemental Table S2). iWUE and *c_i_*/*c_a_* were strongly negatively correlated, as expected for traits that are the result of closely related mathematical expressions. However, trait relationships and genotype-to-phenotype results for the two traits included unique features. So, to avoid excluding potentially valuable information they were both retained in the analysis. Genetic and phenotypic correlations for iWUE were consistent with those for *c_i_*/*c_a_*, except iWUE was also significantly genetically correlated with *A_N_* (*r* = −0.53), and the genetic correlation between iWUE and SD was just outside the significance threshold (Figure 2; Supplemental Table S2). SD was also significantly positively correlated with height on a genetic basis (*r* = 0.38) and phenotypic basis (*r* = 0.08). Height was presented as adjusted genotypic means calculated from a mixed model incorporating data from both 2016 and 2017 because results were so highly correlated between years (*r* = 0.94; Supplemental Figure S4. Meanwhile, SD was phenotypically correlated with SLA (*r* = −0.08).

The associations observed when SD, SLA, and agronomic traits were measured in 2016 were generally consistent with those reported above for 2017, although phenotypic correlations were generally stronger in 2016 (Figure 2; Supplemental Table S2), consistent with the wider range of trait values observed in the absence of drought stress (Figure 3).

While there were significant genetic correlations between data from 2016 versus 2017 for SLA (*r_g_* = 0.78) and SD (*r*_g_ = 0.87), phenotypic correlations between years were weaker (SLA *r_p_* = 0.46, SD *r_p_* = 0.36) and plasticity in response to drought stress was clearly apparent. Under the drought conditions of 2017, SD was 40% lower on average than in wetter conditions of 2016 (Figure 3A). Meanwhile, SLA was 24% greater, on average, in 2017 than in 2016 (Figure 3B). The relative change in SD between growing seasons varied from −9% to −47% and was phenotypically correlated with SLA in 2016 (*r_p_* = −0.10), biomass production (*r_p_* = 0.11), and height (*r_p_* = 0.15; Figure 2). The relative change in SLA between growing seasons varied from −2% to +34% and was phenotypically correlated with the relative change in SD (*r_p_* = 0.15) between growing seasons as well as biomass (*r_p_* = 0.19), height (*r_p_* = 0.37), SD in 2016 (*r_p_* = 0.10), *g_s_* (*r_p_* = −0.011), and iWUE (*r_p_* = 0.07; Figure 2; Supplemental Table S2).

### Genetic basis of WUE associated traits

**Figure 4:**
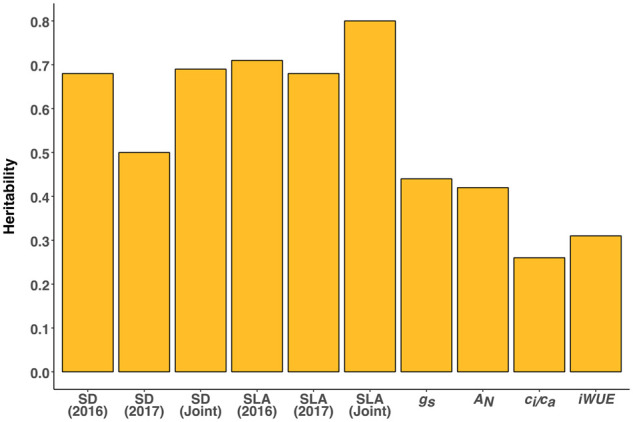
Bar plot of heritabilities of SD, SLA, *g_s_*, net photosynthesis (*A_N_*), *c_i_*/*c_a_*, and iWUE. Models combining individual and joint yearly data were used to estimate heritability for SD and SLA. Gas exchange traits were only measured in 2017.

Generalized heritability was relatively high for SD and SLA (Figure 4). However, the heritability for SD in 2017 (0.50) was lower than in 2016 (0.68) and for SD-joint (0.69). In contrast, heritability for SLA-joint (0.80) was greater than for the individual years of 2016 (0.68) and 2017 (0.71). Leaf-level gas exchange traits demonstrated low to moderate heritability (*g_s_* = 0.44, *A_N_* = 0.42, iWUE = 0.31, *c_i_*/*c_a_* = 0.26).

**Figure 5:**
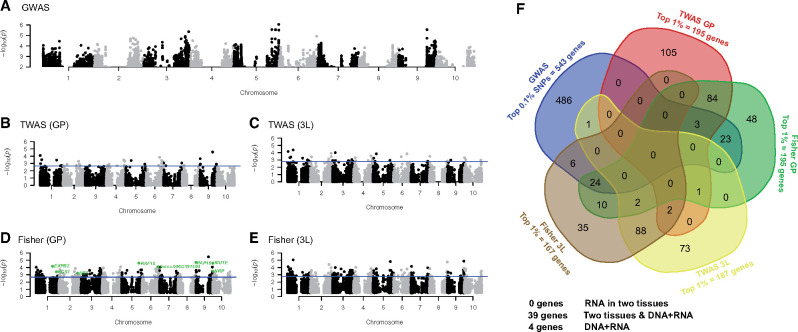
Mapping for SD in 2016. A, GWAS; (B) TWAS in GP tissue; (C) TWAS in the 3L; (D) Fisher’s combined test results in GP tissue; (E) Fisher’s combined test in 3L tissue; and (F) a five-way Venn diagram highlighting where genes within the top sets of all mapping approaches for SD-2016 are consistently identified. B–E, Blue lines indicate the threshold for the genes with the top 1% of −log_10_ (*P*-values). Genes with known or putative roles in stomatal development are highlighted in green. Supplemental Tables S8–S10 for gene lists related to each test, tissue, year, and trait.

**Figure 6:**
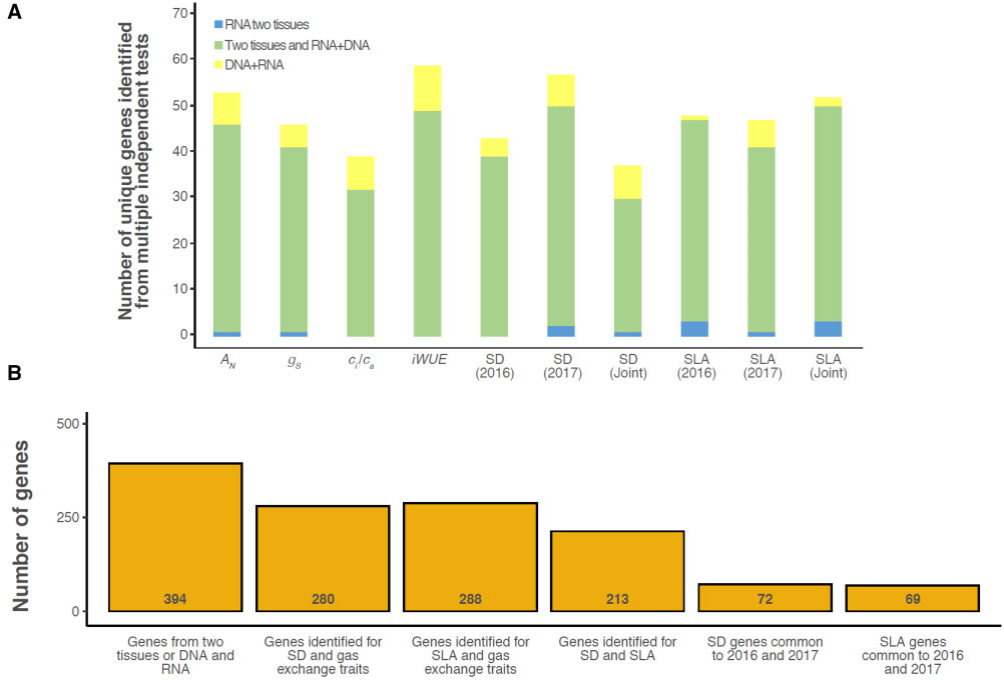
The number of common and unique genes identified via independent tests. A, Barplot of the number of unique genes identified with higher confidence as potentially underlying variation on a trait-by-trait basis for net photosynthesis (*A_N_*), *g_s_*, *c_i_*/*c_a_*, iWUE, SD, and SLA. Higher confidence genes were defined as those identified from multiple tests representing independent evidence from either: TWAS only, but in both tissues (blue fill); Fisher’s combined test and/or TWAS in both tissues (green fill); or GWAS plus TWAS or Fisher’s combined test in one tissue (yellow fill). B, Barplot of the number of unique genes consistently identified in multiple independent tests across different traits or growing seasons. For reference, the total number of unique genes identified by a parallel trait by trait approach (394, see A) is presented in the first bar. See Supplemental Tables S8–S10 for gene lists related to each test, tissue, year, and trait.

For all traits, some regions of the genome contributed more than others to the observed phenotypic variation. A three-tiered approach for genetic mapping was used to identify candidate genes underlying the variation observed for the WUE-associated traits under study. Adjusted genotypic means from each linear mixed model for each trait were used for a GWAS (e.g. Figure 5A). The genes within linkage disequilibrium (LD) of the most statistically significant 0.1% of GWAS SNPs (Supplemental Table S3) were then identified (Supplemental Table S4). The number of independent genes identified per trait varied from 475 for SLA in 2016 to 656 for *A_N_* in 2017 (Figure 5F;Supplemental Figures S5–S13). The top 1% of gene transcripts that had the most significant associations with a given trait (Supplemental Table S5; Figure 6, B and C; Supplemental Figures S4–S13) were identified via TWAS. TWAS was performed independently using RNAseq data from two tissue types (Supplemental Figure S14): (1) the shoot growing point (GP; 195 genes per trait) and (2) the developing third leaf (3L; 167 genes per trait). GWAS and TWAS *P*-values were integrated via Fisher’s combined probability test to identify candidate genes that the two orthogonal tests combined suggested to contribute to the observed phenotypic variation (Supplemental Table S6; Supplemental Figures S4–S13; Figure 6, D and E). We also extracted the top 1% most significant genes identified via the Fisher’s combined approach, such that for each trait, five independent lists of top genes were generated, that is, those from GWAS, TWAS-GP, TWAS-3L, Fisher-GP, and Fisher-3L.

Candidate genes identified in two or more independent tests are less likely to be false positives, that is, more likely to be associated with genetic variation in the traits of interest. Therefore, consistency in results was tested: (1) across test types for a single trait (Figure 6A) and (2) across key trait groups, years, or test-types (Figure 6B). Between 37 and 59 candidate genes were identified with high confidence for a given trait, based on being identified in at least two independent tests (Figure 6A;Supplemental Figures S4–S12; Supplemental Tables S7 and S8). This criterion was most consistently met when the tests integrated data about trait associations with both DNA sequence variation (i.e. GWAS) and RNA transcript abundance variation (i.e. TWAS and/or Fisher’s combined GWAS–TWAS). The importance of transcript abundance variation to this end was observed for developing leaf (3L) and GP tissues (Figure 6B). This highlights the value of TWAS for supporting moderate GWAS signals. For example, 47 genes in total met these criteria for SD in 2016 (Figure 5F) by being consistently identified by both Fisher’s tests (10 genes), both Fisher’s tests plus one TWAS test (2 genes), both Fisher’s test plus the GWAS (24 genes), or a Fisher’s test and both TWAS tests (2 + 1 genes). In addition, a moderate number of high confidence genes was identified when the tests integrated data about trait associations with DNA sequence variants and RNA transcript abundance in a single tissue (Figure 6A). For example, four genes met this criterion for SD in 2016 by being consistently identified by the GWAS and TWAS (one gene) or GWAS, TWAS, and Fisher’s test (three genes) for a given tissue (Figure 5F).

**Figure 7:**
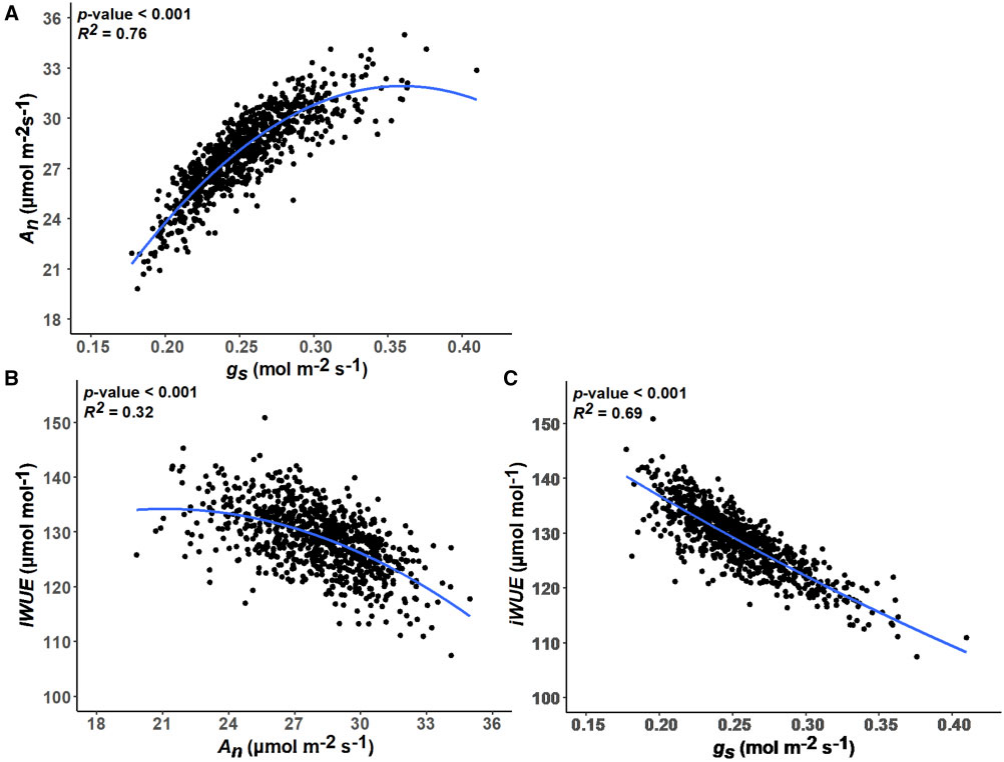
Relationships between gas exchange-related traits. All data are adjusted means. A, Relationship between *g_s_* and net photosynthesis (*A_n_*). B, Relationship between *A_n_* and iWUE. C, Relationship between *g_s_* and iWUE. For each relationship, a second-order polynomial model regressing *y* on *x* is fitted (blue) and the associated standard error of the model is highlighted (gray ribbon). The associated *P-*value significance threshold and *r^2^* values for each model are inset.

The smallest number of “high confidence” genes was found by being identified in TWAS tests for both tissues, without evidence for genotype to phenotype associations from GWAS (Figure 7A). For example, two genes met this criterion for SD in 2017 (Supplemental Figure S5F). These patterns were consistent for all the traits tested. When compiled across all the leaf traits, these multiple independent tests identified 394 unique candidate genes for associations with trait variation for RNA plus DNA, or from both tissues where the transcriptome was tested (Figure 7B;Supplemental Tables S7 and S8). We utilized genome-wide SNP data from a partially matching set of sorghum lines (284/499; Lozano et al.,2021), to explore the presence of SNPs within the coding sequences of these candidate genes as evidence of existing polymorphisms that may be regulating observed phenotypic variation. It revealed that at least 275 of the 394 highest confidence candidate genes contain one or more SNPs that drive a nonsynonymous substitution leading to a predicted change in protein function by the sorting intolerant from tolerant (SIFT) score (Supplemental Table S9).

Candidate genes were also consistently identified by two or more tests that spanned key trait groups (Figure 7B). For example, 213 genes were independently identified by tests for both SD and SLA. 280 genes were independently identified by tests for both SD and photosynthetic gas exchange traits. 288 genes were independently identified by tests for both photosynthetic gas exchange traits and SLA. Comparing across independent tests in separate growing seasons, 72 genes associated with variation in SD were consistently identified in 2016 and 2017. In contrast, 69 genes associated with variation in SLA were identified in both years.

In at least 75 cases, the putative orthologs in Arabidopsis of candidate genes identified here are annotated by the arabidopsis information resource (TAIR; www.arabidopsis.org) as having some function related to leaf development or WUE (Supplemental Table S10 and references therein). For example, AT3G06120 (MUTE) shares the greatest sequence similarity with Sobic.009G260200 identified for SD and encodes a bHLH protein that controls meristemoid differentiation during stomatal development (Pillitteri et al., 2007). AT1G51660 (MAPK4) is most similar to Sobic.004G323600 identified for *g_s_* and is a disease resistance protein involved in ABA-regulated stomatal movements (Hettenhausen et al., 2012; Witoń et al.,2016). AT4G00430 (PIP1;4) is most similar to Sobic.006G176700 identified for *A_N_* and is a CO_2_ transporter involved in photosynthetic metabolism (Li et al., 2015). At least 46 of these 75 candidate genes contain one or more SNPs that drive a nonsynonymous substitution leading to a predicted change in function by the SIFT score (Supplemental Table S19).

## Discussion

The tradeoff between carbon gain and water use is a fundamental constraint for crop productivity and environmental resilience (Bailey-Serres et al., 2019; DeLucia et al., 2019; Leakey et al., 2019). More specifically, improving WUE is recognized as a means to enhance the utility of sorghum as a biofuel feedstock (Mathur et al., 2017; Meki et al., 2017). Nevertheless, understanding of genetic variation in traits that underlie iWUE in C_4_ grasses is poor even after more than a century of WUE research (Briggs and Shantz, 1917; Leakey et al., 2019). This study successfully met the goal of developing an integrated approach for rapid iWUE phenotyping. It used these technical advances to provide one of the largest and most comprehensive investigations of genetic and environmental variation in leaf traits that influence WUE, that is, *g_s_* (Hatfield and Dold, 2019; Leakey et al., 2019), *A_N_* (Hatfield and Dold, 2019; Leakey et al., 2019), SD (Bertolino et al., 2019), and SLA (Zhang et al., 2009, 2015). A novel element of that investigation was integrating GWAS, TWAS, and predictions of deleterious variants to identify candidate genes that can be further studied to understand and improve iWUE in sorghum and other C_4_ crops.

### Rapid phenotyping

Traditional assessments of traits relating to leaf gas exchange and stomatal patterning are time and labor-intensive. For example, measuring the light-saturated gas exchange of individual leaves can take >30 min (Ortiz et al., 2017; Qu et al., 2017), and manually peeling leaf epidermal samples and counting stomata via light microscopy is slow (Yates et al., 2018). Consequently, these traditional approaches are not readily amenable to large-scale assessments of genetic variation. A high-throughput phenotyping pipeline (Supplemental Figure S15) was developed by integrating: (1) a rapid method of measuring leaf-level gas exchange (Supplemental Figure S16; Choquette et al., 2019); (2) rapid scanning of abaxial leaf surfaces and automated stomatal counting (Figure 1;Supplemental Figure S3); and (3) sampling for SLA. Over 200 leaves were processed per day, facilitating phenotyping of 869 accessions replicated across two field trials in each year. This was a substantial gain in scale over previous experiments looking at similar traits in isolation (Taylor et al., 2016; Ortiz et al., 2017; Herritt et al., 2018; Lü et al., 2018; Yates et al., 2018). Our automated approach for determining SD was validated by comparisons to ground truth data (Figure 1C). Computer-based measurement of SD in 33,355 FOV was approximately 80 times faster than the counting of stomatal complexes by humans. Importantly, the efficacy of the method across a wide range of genetic and environmental variations in epidermal leaf anatomy was highlighted by the moderate-to-high heritability of SD (Figure 5). These heritability estimates were similar or higher than those previously reported (Dittberner et al., 2018). A variety of machine learning methods have been developed that can identify stomata in images (e.g. Fetter et al., 2019; Sakoda et al., 2019), but demonstrations of their applicability to large-scale genetic studies of the measured trait are rare (Dittberner et al., 2018) to nonexistent, depending on the species. Overall, this work, along with Xie et al. (2021) and Bheemanahalli et al. (2021), demonstrates the utility of optical tomography and computer vision as tools that can meet the potential for accelerating biological discovery in cereal crops.

### Genetic variation in SD, *c_i_*/*c_a,_* iWUE, *A_N_*, and *g_s_*

We detected a positive association between *A_N_* and *g_s_* (Figure7. This is consistent with previous studies of diverse germplasm in C_4_ crops, such as maize (Choquette et al., 2019), sugarcane (Inman-Bamber et al., 2016), and switchgrass (Taylor et al., 2016). And, it affirms that accessions with greater *g_s_* achieve greater rates of *A_N_*, despite sorghum having a biochemical pump concentrating CO_2_ around Rubisco in the bundle sheath cells. However, the nonlinear nature of the relationship also indicates diminishing returns from greater *g_s_* in terms of *A_N_*, leading to lower iWUE among accessions with the greatest *g_s_*. Selection for greater productivity in other crops has been associated with greater *g_s_* and water use (Roche, 2015; Koester et al., 2016). Repeating the same strategy would not be desirable in sorghum, assuming that high productivity under water-limited conditions is a priority. Notably, the genetic variation observed in iWUE (or the closely related *c_i_/c_a_*) was more a factor of variation in *g_s_* (Figure 7C) than in *A_N_* (Figure 7B). Taken together, these results demonstrate that enhanced iWUE is achieved either through low *g_s_* or through coupling high *A_N_* with moderate *g_s_*. While in the past, it was suggested that WUE across C_4_ species was almost invariant (DeLucia et al., 2019), this study builds on work in sugarcane (Inman-Bamber et al., 2016) to suggest that meaningful variation does exist. Our generalized estimates of heritability for *A_N_* and *g_s_* (Figure 4) were similar to those estimated in a recent survey of the same traits in a smaller panel of grain sorghum accessions (Ortiz et al., 2017) and sufficiently high to justify targeting them as traits for selection. But, efforts to improve WUE in sorghum via the direct selection on iWUE may inadvertently limit *A_N_* in the same way as previously observed in C_3_ crops (Condon et al., 2004; Leakey et al., 2019). So, understanding a broader set of component traits that influence iWUE will be valuable.

Consistent with theoretical expectations and prior observations in grass crops (Miskin et al., 1972; Muchow and Sinclair, 1989), *c_i_*/*c_a_* was genetically correlated with SD (*r* = 0.41; Figure 2; Supplemental Table S2). However, iWUE, which is closely mathematically related to *c_i_*/*c_a_*, was only marginally genetically correlated with SD. When environmental sources of variance were accounted for in addition to genetic factors, there were no significant phenotypic correlations between SD and leaf gas exchange traits across the diverse panel of sorghum accessions (Figure 2). The significant fraction of genetic variation in *c_i_*/*c_a_* explained by SD across the diverse accessions supports the contention that SD is a viable target for improvement in C_4_ crop performance. Grain sorghum accessions selected for high or low SD alleles at a single locus display corresponding variations in *g_s_* (Bheemanahalli et al., 2021). Transgenic approaches to reducing SD have reduced *g_s_* and increased iWUE in a number of crops (Wang et al., 2016a; Hughes et al., 2017; Dunn et al., 2019; Mohammed et al., 2019). Therefore, advancing the understanding of genes and trait associations underpinning SD and iWUE has the potential to aid crop improvement efforts.

But, the trait relationships reported here also highlight the complex mix of intrinsic and environmental factors that affect WUE in a field-grown crop (Leakey et al.,2019). For example, detecting an association between SD and *g_s_* may be complicated by a strong tradeoff between SD and stomatal size (Xie et al., 2021). In maize, the width and length of stomatal complexes were significantly correlated with leaf gas exchange traits (Xie et al., 2021). And, in rice, stomatal length has been observed to positively correlate with *g_s_* across rice accessions, where SD did not (Ohsumi et al., 2007). So, additional work is needed to fully understand how multiple aspects of stomatal patterning, anatomy and opening combine to influence iWUE.

### Genetic and environmental variation in SD and SLA

iWUE had a strong positive genetic correlation with SLA (Figure 2; Supplemental Table S2, suggesting that thicker leaves have reduced iWUE. Consequently, it appears that there is an important tradeoff between maximizing photosynthesis through leaf structure while minimizing associated water loss. When considering this tradeoff, it is worth noting that iWUE, but not *A_N_*, demonstrated a significant positive genetic correlation with total above ground biomass (Figure 2; Supplemental Table S2). This indicates that selecting to improve iWUE may not compromise yield in biomass sorghum.

SD was substantially lower in the dry growing season of 2017 than the wet growing season of 2016 (Figure 3A). The morphology and patterning of stomata can be modified in developing leaves in response to environmental cues (Lake et al., 2001; Casson and Hetherington, 2010). Lower SD would tend to limit water loss via transpiration under dry conditions, consistent with many other mechanisms that operate to sense soil water content and conserve water (Franks and Farquhar, 2001). However, the response of SD to limiting water supply varies among studies, species, and with the intensity of drought stress (Quarrie and Jones, 1977; Hamanishi et al., 2012; Sakurai et al., 1986). So, the consistent direction of response toward lower SD under drought conditions experienced in the field by this diverse sorghum population is noteworthy.

SLA was substantially greater, on average, in the drier growing season (Figure 3B), indicating an overall reduction in leaf thickness or density. SLA is well documented to demonstrate remarkable phenotypic plasticity in response to environmental stimuli (Hulshof et al., 2013; Wellstein et al., 2017). Depending on the prevailing conditions, SLA can be coupled to important functional traits, such as photosynthesis and growth rate (Pengelly et al., 2010; Liu et al., 2016; Wellstein et al., 2017; Gonzalez-Paleo and Ravetta, 2018), as well as water-use strategies and WUE (Wang et al., 2013; Scartazza et al., 2016).

Greater SLA allows the same leaf area to develop with a lower investment of carbon resources. Therefore, it is possible that the greater SLA observed under drought conditions facilitated greater investment in other carbon sinks, such as root growth (Wellstein et al., 2017), thereby improving water uptake (Tardieu et al., 2017). This hypothesis is supported by studies that have observed positive correlations between SLA and root foraging ability and specific root length in grass species under conditions of reduced water availability or resource limitations (Pérez-Ramos et al., 2013; Freschet et al., 2015). However, further studies specifically testing this hypothesis in sorghum are required. The increase in SLA will likely have limited leaf-level carbon fixation (Xu and Zhou, 2008; Gonzalez-Paleo and Ravetta, 2018), due to a reduction in the thickness of the chloroplast-rich palisade mesophyll (Gonzalez-Paleo and Ravetta, 2018; Gotoh et al., 2018). This is reflected in the significant negative association observed between *A_N_* and SLA as well as *g_s_* and SLA in 2017 (Figure 2). Despite this reduction in leaf-level *A_N_*, in a canopy with high leaf area index (LAI) like that in biomass sorghum, the side-effect of increased light transmission into the canopy may ameliorate losses in carbon gain at the canopy level (Evans and Poorter, 2001; Liu et al., 2016).

Plant height and biomass were positively correlated with the percentage change in trait values between growing seasons for both SD and SLA (Figure 2; Supplemental Table S2). While many factors could contribute to this relationship, the most parsimonious explanation would be that more productive accessions generally have the greatest demand for water, exhaust available resources to the greatest extent, and then demonstrate the greatest plasticity in anatomy and physiology required to avoid further drought stress. Further work is needed to understand the adaptive value of the observed plasticity for maintenance of productivity when water is limiting. It will also be important to learn if transgenic approaches to increasing iWUE via lower SD constrain plasticity under drought stress. Photoperiod-sensitive sorghum provides a special opportunity to investigate these mechanisms of plastic response to drought because the continued production of new leaves throughout the growing season allows the plant to respond to variation in drought stress to a greater degree than grain sorghum, which has a fixed canopy after the floral transition.

At the genetic level, understanding of the mechanisms determining SD and SLA has not been integrated. But, accessions displaying the greatest plasticity in SD tended to be more plastic in terms of SLA as well (Figure 3). A causal link between the two traits was not explicitly tested in the current study. But, the observed correlations (Figure 2; Supplemental Table S2) are consistent with previous reports of greater leaf thickness enhancing the capacity for reductions in SD under drought (Galmés et al., 2007; Xu and Zhou, 2008). SLA in 2017 negatively associated with *A_N_* (Figure 2; Supplemental Table S2), suggesting that thicker leaves have higher rates of carbon fixation (Wright et al., 2001), which is in line with previous findings and reflects how enhanced mesophyll cell height can promote CO_2_ uptake (Oguchi et al., 2005; Terashima et al., 2011; Coneva and Chitwood, 2018). Theory dictates that greater maximum *g_s_*, via greater SD or stomatal size, along with other aspects of hydraulic capacity in leaves, should support greater exchange of water vapor for CO_2_ to be assimilated through photosynthesis (Dow et al., 2014). This emphasizes the need to better integrate an understanding of relationships of epidermal patterning with the anatomy and function of the leaf as a whole. Consequently, candidate genes associated with variation in both SD and SLA may be of special interest (Supplemental Tables S7 and S8).

### Combining GWAS and TWAS to identify candidate genes

The results of both GWAS and TWAS reinforced the prevailing understanding that iWUE, and associated leaf traits, are complex and polygenic (e.g. Des Marais et al., 2014; Ortiz et al., 2017). As a consequence, and in common with many GWAS studies on a diverse range of traits (Zhu et al., 2008; Ortiz et al., 2017; Kremling et al., 2019), many moderate associations were detected. This is consistent with individual alleles of small or moderate effect sizes segregating at moderate or low frequencies, respectively. In such cases, extra information is needed to avoid reporting false-positive associations and boost confidence in the identification of candidate genes. This study provides a demonstration of the concept tested by Kremling et al. (2019), where GWAS and TWAS are combined to achieve this goal. A total of 394 unique candidate genes were identified for the set of 10 leaf traits studied. To be included in this list, a gene had to be identified for a given trait in multiple independent tests for either: (1) associations of trait variation with both RNA and DNA or (2) associations of trait variation with transcript abundance in both tissue sample types (Figure 5;Supplemental Figures S5–S13; Supplemental Tables S7 and S8). This was the case for 37–59 genes per trait, with 80 genes meeting these criteria simultaneously for 2 or 3 traits. Detailed examination of the results on a trait-by-trait basis revealed the greatest consistency in results coming from the use of Fisher’s combined test to integrate information from the GWAS with TWAS. But, there were examples where the same gene was identified from TWAS performed separately on transcriptome data from both tissue sample types (growing tip versus developing the 3L). In addition, confidence in the identification of other genes was greater because they were independently identified in both growing seasons (72 genes for SD and 69 genes for SLA), or they were identified for multiple traits resulting from independent measurements. The consistency across results for different types of traits in 2017 (213 genes for SD plus SLA; 280 genes for SD plus gas exchange traits; 288 genes for SLA plus gas exchange traits; Figure 6B) was higher than for across growing seasons. But, this does not seem surprising given the difference in water availability between the two years and the potential for genotype × environment interactions. Confirmation of a role for these genes in driving variation in iWUE and related traits will still require a reverse genetics approach performed on a gene-by-gene basis. But, a substantial number of the candidate genes identified are putative orthologs of genes in *A. thaliana* that have functions linked in some way to iWUE, *A_N_*, *g_s_*, or leaf development and anatomy (Supplemental Table S10). The preponderance of genes with associations between trait variation and transcript abundance may indicate that regulatory variation is a more common driver of genetic variation than sequence variants. It is worth noting that TWAS was performed using transcriptome data generated from plants in controlled conditions, as opposed to the field where phenotyping for GWAS was performed. Additionally, tissue sampling at both stages (3L and GP) was limited to a single time point, which is an important consideration in the view of the diurnally dynamic and environmentally sensitive nature of the transcriptome. Despite these potential limitations, the molecular control of the physiological processes of interest is well conserved, which is reflected in the overlap of genes identified by association to RNA and DNA.

### Candidate genes underlying variation in gas exchange and SD

Given the genetic correlation between SD and *c_i_*/*c_a_* observed in sorghum and evidence that manipulating SD can improve WUE in other species (Bertolino et al., 2019), the candidate genes underlying SD identified in this study represent a starting point for future crop improvement to improve iWUE. Moreover, elucidating these candidates for their role in regulating SD may form the foundations of understanding stomatal development pathways in C_4_ grasses.

Genes identified by the combined GWAS/TWAS for SD in this study and that have putative orthologs with demonstrated roles in stomatal development included *3-KETOACYL-CoA SYNTHASE 1* (*KCS1*), which controls stomatal patterning relative to CO_2_ concentration (Gray et al., 2000), and *HOMEOBOX-7* (*HB-7*), which regulates stomatal size relative to water availability (Ré et al., 2014; Figure 5; Supplemental Table S10). Putative SD candidates also included a cell wall expansin-type protein EXPANSIN B2 (*EXPB2*; Marowa et al., 2016), an ABA-sensitive MAP KINASE (*RAF10*; Lee et al., 2015), the *PAP10* purple acid phosphatase (Hepworth et al., 2016), and an asparagine-rich protein (*NRP*) that is documented to positively regulate the expression of *CRYPTOCHROME 2* (*CRY2*; Zhou et al., 2017), a blue light receptor which in turn increases stomatal index (Kang et al., 2009; Figure 5;Supplemental Table S10). Further genes with known roles in stomatal development identified via GWAS/TWAS hits for multiple leaf physiological traits included *ALTERED MERISTEM PROGRAM 1* (*AMP1*; Shi et al., 2013; López-García et al., 2020), *ATPASE E1* (*ATE1*; Movahedi, 2013; Vicente et al., 2019), and *REVERESAL OF THE DET PHENOTYPE 5* (*TED5*; Tossi et al., 2014; Zoulias et al., 2020). *EPF*2 was identified via GWAS for *A_N_* and contained a putative deleterious mutation (Supplemental Table S10). *EPF2* regulates stomatal development in response to CO_2_ to coordinate gas exchange and CO_2_ transport to the mesophyll (Engineer et al., 2014; Dow et al.,2017), thereby having a putative role in regulating *A_N_*. Additionally, through mapping for SD, we identified multiple genes with putative and known roles in stomatal behaviur, for example, the *CHLORIDE CHANNEL C* (*CLC-C*) anion transporter (Jossier et al., 2010), and ABA responsiveness, for example, *FAR1-RELATED SEQUENCE 5* (*FRS5*; Ma and Li, 2018), which represent interesting targets for further study. Many of these candidate genes were demonstrated to contain multiple nonsynonymous and deleterious SNPs that would be predicted to lead to substantial functional variation across sorghum (Supplemental Table S10). For example, *KCS1* was observed to contain six nonsynonymous mutations. One of these is classified as deleterious, with a further two being only just above the threshold required for being considered deleterious (0.07 and 0.05; Supplemental Table S10). Similarly, *AMP1* also contained more than 10 nonsynonymous mutations, three of which were deleterious (Supplemental Table S10). Overall, 275 of the 394 genes identified with the highest confidence through the integration of GWAS/TWAS contained nonsynonymous/missense variants that are predicted to have deleterious impacts. This increases the likelihood that the genes identified could cause the observed phenotypic variation. However, the current results estimate this in a conservative fashion since variant calls were not available for the entire sorghum population that was phenotyped. In addition, it is important to acknowledge the existence of tolerated nonsynonymous mutations, as well as the rarer deleterious mutations, within the candidate genes since these may be under selection, be nonneutral and have phenotypic/fitness consequences (Ingvarsson, 2009, Lebeuf-Taylor et al., 2019, Lozano et al.,2021; Wei, 2020). Their presence highlights the potential for selection for variation at these loci for fine-tuning stomatal development within sorghum.

In terms of genes identified underlying variation in *g_s_*, but not *A_N_*, the *Enhanced Downy Mildew Resistance 3* (*EDM3*) protein identified via GWAS and TWAS (Supplemental Table S10) is a promising candidate (Supplemental Table S10). Stomatal physiology is intimately linked to disease resistance, especially with respect to mildew and rust pathosystems, where associated disease resistance proteins can regulate stomatal closure (Prats et al.,2007). Indeed, a recent poplar association study demonstrated a substantial enrichment for disease resistance proteins when mapping for stomatal traits (McKown and Bergmann, 2020). *EDM3* holds particular promise since it forms a complex with *EDM2*, where both proteins have been demonstrated to be required for the prevention of DNA hypermethylation at leucine-rich repeat receptor-like kinases (LRR-RLK) that facilitate immunity (Lai et al., 2019). Indeed, mutations to *EDM2* have been demonstrated to result in hypermethylation of the *ERECTA* family of LRR-RLK genes, which result in stomatal defects (Wang et al., 2016b). Moreover, *EDM3* is noted to contain 12 nonsynonymous mutations, three of which are classified as deleterious, thereby highlighting its potential for imparting variation in stomatal behavior across sorghum. Similarly, a further uncharacterized LRR-RLK (Sobic.001G459500) was also identified via GWAS and TWAS for *g_s_*, but not for *A_N_*, where confidence in its role for *g_s_* is imparted by the presence of multiple nonsynonymous and two deleterious coding sequence mutations across sorghum natural variants (Supplemental Table S10). *MAP KINASE 4* (*MAPK4*) is another gene identified via GWAS for *gs* that is known to be involved in the response to pathogen recognition (Berriri et al.,2012). Furthermore, evidence from Aspen (*Populus termuloides*; Witoń et al., 2016) and Caribbean agave (*Agave angustifolia*; Sara et al., 2020) demonstrate a role for *MAPK4* in the regulation of *g_s_* and stomatal development, which is in line with the identification of *MAPK4* via TWAS for SD in GP tissue also. *MAPK4* was not observed to contain any deleterious SNPs, but the presence of two nonsynonymous SNPs highlights the potential for functional variation at *MAPK4* across sorghum.

*Chlororespiratory Reduction 23* was identified via all mapping approaches for *A_N_* (Supplemental Table S10) and is known to be critical for stabilizing the chloroplast NAD(P)H dehydrogenase complex, thereby facilitating photosynthetic electron transport (Shimizu et al., 2008). The importance of this complex in controlling the observed variation in *A_N_* was further highlighted by the identification of the *Alternative Oxidase 1A* (*AOX1a*) gene via multiple *A_N_* mapping approaches (Supplemental Table S10). *AOX1a* is well demonstrated to play a key role in electron transport and balancing the redox state of cellular NAD(P)H pools, thereby facilitating efficient photosynthetic functioning (Vishwakarma et al., 2014; Podgórska et al., 2020). Combined GWAS and TWAS for *A_N_* also identified genes with demonstrated roles in chloroplast biosynthesis. For example, *Phytoene Desaturase 3* (*PDS3*; Supplemental Table S10) is a key component of retrograde signaling during chloroplast development. Indeed, *pds3*-mutants display an albino phenotype (Foudree et al., 2010). *PDS3* was also identified via mapping for *g_s_* and SD, which is interesting since *PDS* genes have been implicated in ABA biosynthesis and the control of stomatal opening (Chao et al., 2014). Interestingly, all six of the nonsynonymous mutations identified within *PDS3* were defined as deleterious (Supplemental Table S10), highlighting how allelic variation here is likely to translate to phenotypic variation. *Phytochrome Interacting Factor 3* (*PIF3*) *w*as identified via mapping for *A_N_* and *g_s_* (Supplemental Table S10). *PIF3* is a light-dependent transcriptional repressor of genes involved in chlorophyll biosynthesis and further photosynthetic processes (Liu et al., 2013). Additionally, the closely related *PIF4* gene has been demonstrated to regulate the expression of *Speechless* (*SPCH*), a master regulator of stomatal development (Casson et al., 2009; Lau et al., 2018), thereby hinting at a possible role in regulating *g_s_*. Additionally, mapping for *A_N_* identified *Plasma Membrane Intrinsic Protein 1;4* (*PIP1;4*; Supplemental Table S10), which is an aquaporin that regulates the permeability of the plasma membrane to CO_2_, thereby mediating CO_2_ transport for photosynthesis (Li et al., 2015). However, it is worth noting that the role of aquaporins in mediating CO_2_ transport more generally is not abundantly clear (Kromdijk et al., 2019); therefore, further functional validation of the role of *PIP1;4* in sorghum, as well as the other genes identified in this study, is required.

## Conclusion

This study demonstrates the application of novel high-throughput phenotyping tools with combined GWAS/TWAS and predictions of deleterious variants to study the genetic basis for a challenging set of complex traits related to iWUE in a model C_4_ crop. In doing so, it revealed heritable variation in multiple traits that selection could act upon to improve performance under water-limited conditions. In addition, it highlights the central role that SLA may play as an allometric trait that is associated with broad genetic and environmental variation in SD, leaf photosynthetic gas exchange, and plant productivity. Lastly, genomic and transcriptomic variation across this diversity set were leveraged to identify multiple candidate genes with known and putative roles for key WUE traits.

## Materials and methods

### Germplasm and experimental design

In total, 869 previously described biomass sorghum (*S. bicolor*) accessions (Valluru et al., 2019; Dos Santos et al., 2020) were used in this study (Supplemental Figure S1; Supplemental Table S1). All lines were grown during 2016 and 2017 across two field sites in Central Illinois (Savoy, IL), where experiments were sown in late May and harvested in late October. Lines were grown according to an augmented block design as reported previously (Valluru et al., 2019; Dos Santos et al., 2020). Briefly, each field site had one complete replication of the field design that consisted of 960 3m-long four-row plots laid out in a 40-row by 24-column arrangement. Each field consisted of 16 incomplete blocks that were augmented with six common accessions. Between row spacing and overall planting, density was targeted at 0.76 m and 270,368 plants ha^−1^, respectively.

### High-throughput leaf-level gas exchange, leaf tissue sampling, and image collection

The youngest fully expanded leaf of two plants randomly selected from the middle two rows of each plot were excised slightly above the ligule between September 5 and 14, 2016. Damaged leaves were avoided. Excised leaves were immediately placed in a bucket, with the cut surface submerged under water. In the laboratory, three 1.6 cm leaf discs were collected from each leaf while avoiding the midrib. Leaf discs were immediately transferred to an oven set at 60°C for 2 weeks. The dry mass of leaf discs was determined, and SLA was calculated as the ratio of fresh leaf area to dry leaf mass (cm^2^ g^−1^). The SLA data collected in 2016 were previously reported (Valluru et al., 2019).

A leaf tissue strip approximately 1cm × 3cm in area was also cut from the adjacent portion of the leaf from where the leaf discs were collected. Leaf strips were marked to distinguish the abaxial side, inserted into 2 mL screw-cap tubes, and flash frozen in liquid nitrogen and stored at −80°C. About 150 leaf strip samples were moved to −20°C during active microscopy. Leaf samples were removed from the −20°C freezer and affixed to a microscope slide using double-sided tape with the abaxial side facing up. The surface topography of leaf surfaces was imaged using two Nanfocus µsurf explorer optical topometers (Nanofocus, Oberhausen, Germany) at ×20 magnification with a standardized area of 800 × 800 µm^2^. The upper and lower *z*-scale limits being set manually for each FOV to ensure all stomata were in focus. The abaxial surface topography was imaged at 4–6 randomly selected points producing 4–6 FOV. Files were saved in the .nms format and automatically transferred to a CyVerse (formerly iPlant) data store (Goff et al., 2011). The custom software tool that automatically counted the stomata in these images is described in the next section.

In 2017, leaf-level gas exchange of all accessions was measured in addition to sampling for SLA and tissue for stomatal imaging. The field was divided into four quartiles based on height measured in previous growing seasons. Each quartile was sampled over a 4- or 5-d period. On each measurement day, 200–232 leaves (two leaves from 100–116 plots) were harvested predawn as described for the 2016 SLA and SD leaf sampling. Upon returning the leaves to the lab, stable rates of light-saturated gas exchange were measured by following the experimental protocol described previously for maize (Choquette et al., 2019). This approach yields rates of gas exchange that match those achieved through in-situ measurements (Leakey et al., 2006). In addition, this approach is preferable since it alleviates the substantial logistical challenge of performing these measurements in the field, and it avoids short-term changes in water potential that occur in the field, and that may limit photosynthesis. Stable rates of net photosynthetic CO_2_*A_N_*, *g_s_*, iWUE, and the ratio of intracellular and atmospheric CO_2_ (*c_i_*/*c_a_*) were obtained by averaging data from the last 2 min of a 4-min autolog program (Supplemental Figure S14). After the measurements of leaf-level gas exchange, the area of the leaf contained within the cuvette was marked and used for sampling for leaf discs and tissues strips for subsequent measurements of SLA and stomatal imaging as described above for the 2016 sampling campaign. A flow chart describing this pipeline is provided in Supplemental Figure S13.

### A machine learning method for automated stomata counting

Image processing and machine learning methods were combined to produce a software tool that automatically detected stomata in 33,355 grayscale images of sorghum leaf surfaces. Constructing the method required a set of training data based on circular image disks 80 pixels in diameter centered where human experts had registered the locations of stomata in many 512×512 raw images. Each 80-pixel disk was subjected to circular fast Fourier transformation (FFT) to produce a radial series of phase and amplitude values that proved to be predictive of stomata. The radial FFT results were recast by principal components (PCs) analysis into a lower dimensional form that served as the feature set used to train nine different machine learning methods. The nine methods were: An Artificial Neural Network, Linear Discriminant Analysis, a Convolution Neural Network, three Generalized Linear models, two Regularization (Ridge and Lasso), and one without, Partial Least Squares Regression, Stepwise Linear Regression, and a Decision Tree. Each method produced a version of the original image in which each pixel value was a probability of that location belonging to a stoma. Next, a fusion process filtered and combined the independent probability maps such that local probability peaks in excess of a height threshold optimally coincided with the locations of human-verified stomata. Specifically, a Nelder–Mead optimization process adjusted the filter and threshold parameters to maximize the agreement between the machine-labeled stomata and the human-identified stomata as quantified by the Matthews correlation coefficient. Supplemental Figure S3 shows an overview of the method. The analyses were implemented in the Matlab programming environment and deployed on a high-throughput computing resource with jobs scheduled by HTCondor (Thain et al., 2005). The machine learning and optimization processes (i.e. layers) were subsequently trained and tuned accordingly.

Each FOV from a stomatal imaging sample produced a stomatal count value. The stomatal count values were divided by the area of the images (0.64 mm^2^) to give SD. The median of the 4–6 SD estimates was calculated for each sample and used for subsequent analyses. To benchmark the efficiency of the automated stomatal counting, we manually counted and estimated SD for randomly selected samples, which represented 1,056 individual FOV. A linear model predicting manually counted SD from automatic SD was subsequently fit (Figure 1C).

### Plant height and biomass measurements

In 2016 and 2017, a single representative plant in each plot was measured for plant height as described and reported previously (Valluru et al., 2019; Dos Santos et al., 2020). For this study, plant height on 105 d after planting was used for comparative analyses since it showed the greatest heritability of all days measured. In 2017, plants were harvested, and above ground biomass was measured and calculated as dry t ha^−^^1^ as previously described and reported (Dos Santos et al., 2020)

### Statistical models and heritability

For each trait, we fitted a linear mixed model using the ASReml-R v4.0 package (Butler et al., 2017). The appropriate model was chosen based on the Akaike information criterion and the diagnostic plots. As different covariables were evaluated along with each phenotype, the final model varied in each case (Supplemental Table S11). The general model used was as follows:

(1) $$y=1\mu +X_{1}t+X_{2}q+Z_{1}s+Z_{2}b+Z_{3}g+Z_{4}ge+e,$$

where **y** (*n* × 1) is the vector of phenotypes for j environments (year × location combination) with $n\;=\;\sum_{i=i}^{j}n_{i}$; **1** (*n* × 1) is a vector of ones; **µ** is the overall mean; **X**_1_ (*n* × j) is the incidence matrix associated with the vector of fixed effect environments **t** (j × 1); **X**_2_ (*n* × v) is the incidence matrix associated with the vector of fixed effect covariates **q** (v × 1; see Supplementary material for details on the number of fixed effects covariates used in each model, if any); **Z**_1_ (*n* × f) is the incidence matrix associated with the vector of random effect set within environment **s** (f × 1) with $s∼\mathrm{MVN}(0,\;I_{f}⊗S)$; **Z**_2_ (*n* × *r*) is the incidence matrix associated with the vector of random block within set within environment effects **b** (*r* × 1) with $b∼\mathrm{MVN}(0,\;I_{r}⊗B)$; **Z**_3_ (*n* × l) is the incidence matrix associated with the vector of random genotype effects **g** (l × 1) with $g∼\mathrm{MVN}(0,\;I_{l}⊗G)$; **Z**_4_ (*n* × w) is the incidence matrix associated with the vector of random genotype-by-environment effects **ge** (*w* × 1) with $ge∼\mathrm{MVN}(0,\;I_{w}⊗K)$; and **e** (*n* × 1) is the vector of residuals with $e∼\mathrm{MVN}(0,\;{⊕}_{i=1}^{j}I_{n_{i}}⊗R_{i})$. The matrices **S**, **B**, **G**, **K**, and **R** are the variance–covariance matrices for set within environment, block within set within environment, genotype, genotype-by-environment, and residual effects, respectively. For each genotype, we obtained predictions from model 1, and these were used for downstream analysis. The generalized heritability was estimated as proposed by (Cullis et al., 2006). Genetic correlations between all pairwise traits were estimated by fitting bivariate models in ASReml-R v4.0 package (Butler *et al.*, 2017) and modeling the genetic effect with correlation structure “corgh” as in Fernandes et al. (2018). The standard error was obtained from this same model and estimated with the delta method. Finally, we fitted this same bivariate model with the “diag” variance-covariance structure. Since the only difference between the structures “diag” and “corgh” is the correlation term, we tested the two models with a likelihood ratio test to obtain a *P*-value for the genetic correlation.

### RNA-sequencing analysis

A subset of the full diversity panel was grown under controlled experimental conditions for 3′ RNA-sequencing (RNA-seq) analysis of genes potentially involved in regulating leaf development, including stomatal patterning. The abundances of transcripts for putative orthologs of known stomatal patterning genes were initially screened across —three to seven separate tissues at each of four developmental stages during the day and night in six accessions. Based on that screen, the base of leaf three and the shoot GP at the 3-leaf stage were targeted for sample collection during the day from a subset of 229 accessions from the full population (Supplemental Figure S14). Samples were processed, and the expression data were generated from the libraries using a pipeline and parameters similar to Kremling et al. (2019). Briefly, reads were trimmed using Trimmomatic (version 0.32) to remove adapter sequences in relation to *Illumina chemistry* and sequencing errors. Next, trimmed reads were aligned to the sorghum reference genome (version 3.1.1) using the splice-aware aligner, Spliced Transcripts Alignment to a Reference (version 2.4.2). Feature counts were then generated using HTSeq (version 0.6.1) from previously generated alignment files. Finally, count normalization was performed using the R package, DESeq2 via size factor estimation.

### Genome-wide association study

For conducting GWAS, we imputed the 100,435 GBS SNPs from (Dos Santos et al., 2020) using as reference panel the whole-genome resequencing dataset of 5,512,653 SNPs published by Valluru et al. (2019). The untyped genotypes were imputed and phased into haplotypes using Beagle 4.1 using a default window size of 50,000 SNPs and an Ne = 150,000. After the imputation, SNPs with allelic R squared < 0.5 and minor allele count below 20 were removed, resulting in a total of 2,327,896 SNPs. Additionally, we pruned SNPs in high LD (*r*^2^ > 0.9) using Plink options “–indep-pairwise 50 10 0.9”. The final dataset consisted of 454,393 SNPs scored in 836 sorghum lines.

The association analysis was conducted using the unified MLM (Yu et al., 2006) implemented in the software GEMMA (Zhou and Stephens, 2012). For that, predicted values obtained from model 1 were normal quantile transformed as per Zhou and Stephens (2014) to guard against model misspecification. We used the Bayesian information criteria (Schwarz, 1978) to select the appropriate number of PCs to account for population structure. We tested models with 0–10 PCs estimated from TASSEL 5 (Bradbury et al., 2007). The best model did not include any PC. For illustrative purposes, we show that not including PCs had no influence on our GWAS results, highlighting how GWAS signals were not associated with population structure in our study (Supplemental Figure S17). Relatedness was controlled for by a kinship matrix obtained from TASSEL 5 using the default method (Endelman and Jannink, 2012).

### TWAS and combined GWAS–TWAS

A TWAS was performed on a subset (229) of the total accessions (Valluru et al.,2019) and conducted using TASSEL (version 5.2.5). Before mapping, covariates were generated from multiple sources. Ten hidden factors were calculated using probabilistic estimation of expression residual (PEER) factors for each individual tissue (Stegle et al., 2012). Additionally, five genetic principal components (PCs) were calculated from prior genotype data (Valluru et al., 2019). Genes that were expressed in at least half of the individual lines were used within each tissue set. A general linear model was fit individually for each phenotype and every combination of expressed gene value across individuals after adjusting for PC and PEER factor covariates.

TWAS-GWAS combined *P*-values were calculated similarly as described by Kremling et al. (2019). Briefly, *P*-values of the top 10% significant GWAS SNPs were assigned to their nearest gene. Assigned GWAS *P*-values were then combined with their respective TWAS *P*-values via the Fisher’s combined test as using the sumlog function within the R package, metap.

To further explore the results of all genetic mapping approaches, we queried commonality between specific gene sets, for example, genes identified for SD and SLA, SD genes common to 2016 and 2017, Genes identified via GWAS and Fisher’s combined test, etc. (Figure 6B). For these comparisons, the total number of possible shared genes between any two gene sets was determined.

### Candidate gene identification

For each GWAS result, the top 0.1% of SNPs based on −log_10_ (*P-*value) were identified. The LD blocks these SNPs associated with were determined, and all genes within these LD blocks or spanning their borders were extracted. LD blocks were estimated based on the method proposed by (Gabriel, 2002) and implemented in PLINK (Chang et al., 2015). For this, we used the option –blocks, with a window of 200 kb and default values for D-prime’s confidence interval (0.7; 0.98). For the TWAS and the combined GWAS-TWAS, the top 1% of genes based on −log_10_ (*P-*value) were identified for each result. This approach of selecting a percentage of the top hits for each method is necessary when comparing results from orthogonal methods (GWAS/TWAS) that are differently powered and structured, and therefore a direct comparison of *P*-values is not appropriate (Kremling et al., 2019). A list of candidate genes with known or putative roles in associated traits was determined based on the overlap between different mapping approaches and/or traits. This combination of methods allows for the detection of weaker effect associations when they are consistently found across multiple independent tests in order to balance the risk of Type-I versus Type-II errors.

For each highlighted candidate gene, we employed the genome-wide data generated by Lozano et al. (2021) to identify SNPs within the coding sequences of these genes. These genome-wide SNP data were generated using 499 lines, of which 286 are common to our study. For each of the reported candidate genes (Supplemental Table S10), we report all associated SNPs and highlight whether they are synonymous or nonsynonymous and their purported effect on protein function based on the SIFT scores (Lozano et al., 2021).

### Additional statistical analysis and figure generation

All further statistical analysis and figure generation were performed within the R environment (R Core Team, 2017). Change in SLA and SD across the two growing seasons was calculated at the accession level as percentage change using adjusted means. SLA increased in all but two accessions across the two years. Thus, the percentage change in SLA was calculated as (2017_SLA_–2016_SLA_)/2017_SLA_×100. SD decreased in all accessions across the two years. Thus, the percentage change in SD was calculated as (2016_SD_–2017_SD_)/2016_SD_×100. Tests for associations between all pairwise trait interactions were performed using adjusted means from all models and Pearson’s product-moment correlation coefficient. Pairwise interactions between specific leaf-level gas exchange traits were further investigated by fitting second-order polynomial equations between traits. Except for Manhattan plots, all figures were generated using the R package ggplot2 (Wickham, 2016). Manhattan plots for all genetic mapping visualization were generated using the R package qqman (Turner, 2017).

### Data availability

The 3-prime RNA-sequencing data (accession number: PRJNA522466) are available at: https://www.ncbi.nlm.nih.gov/bioproject/PRJNA522466/. Genotyping-by-sequencing data are available at: https://doi.org/10.5281/zenodo.5019227. Phenotypic data are available as part of the supplemental material (Supplemental Table S12). Optical tomography images from this article can be found in the Illinois Data Bank under: https://doi.org/10.13012/B2IDB-1411926_V1.

## Supplemental data

The following materials are available in the online version of this article.

**Supplemental Figure S1**. Map showing the point of origin of all accession employed in this study.

**Supplemental Figure S2**. Temperature and precipitation during growing seasons every 5 d of year.

**Supplemental Figure S3**. Overview of stomatal counting machine learning method.

**Supplemental Figure S4**. Plant height correlation between growing seasons.

**Supplemental Figure S5**. Mapping for SD in 2017.

**Supplemental Figure S6**. Mapping for SD (joint year model).

**Supplemental Figure S7**. Mapping for SLA in 2016.

**Supplemental Figure S8**. Mapping for SLA in 2017.

**Supplemental Figure S9**. Mapping or SLA (joint year model).

**Supplemental Figure S10**. Mapping for stomatal conductance.

**Supplemental Figure S11**. Mapping for photosynthesis.

**Supplemental Figure S12**. Mapping for iWUE.

**Supplemental Figure S13**. Mapping for the ratio of intracellular to ambient CO_2_.

**Supplemental Figure S14**. Tissue harvested for RNA-seq analyses.

**Supplemental Figure S15**. Phenotyping pipeline.

**Supplemental Figure S16**. Example of gas exchange data collection.

**Supplemental Figure S17**. Impact of inclusion of principle components for GWAS.

**Supplemental Table S1**. List of accessions comprising this study.

**Supplemental Table S2**. Pairwise correlations between traits.

**Supplemental Table S3**. Top 0.1% SNPs identified through GWAS for each trait.

**Supplemental Table S4**. Genes within LD of the top 0.1% SNPs identified through GWAS for each trait

**Supplemental Table S5**. Top 1% of genes identified through TWAS for each trait and each tissue type

**Supplemental Table S6**. Top 1% of genes identified through Fisher combined GWAS–TWAS for each trait and each tissue type.

**Supplemental Table S7**. Tall version of genes identified in the top sets of all mapping approaches.

**Supplemental Table S8**. Wide version of genes identified in the top sets of all mapping approaches.

**Supplemental Table S9**. Presence of deleterious SNPs within the 394 high confidence genes.

**Supplemental Table S10**. List of candidate genes with known or putative roles associated to the traits for which they were identified.

**Supplemental Table S11**. Parameters included in the mixed linear model for the analysis of SD, *g_s_*, *A_N_*, *c_i_*/*c_a_*, iWUE, and SLA.

**Supplemental Table S12**. Adjusted genotypic means for all traits comprising this study.

## Supplementary Material

kiab346_Supplementary_Data

## Notes

A.D.B.L., E.P.S., P.J.B., M.A.G., and E.S.B.: designed the research; J.N.F., S.BF., D.A., A.D., and P.S.: performed the research; N.D.M. and E.S.P.: provided computational tools for image analysis; J.N.F., S.B.F., B.M., R.V., R.L., and A.D.B.L.: analyzed the data. J.N.F. and A.D.B.L. wrote the article with input from all authors.

The author responsible for the distribution of materials integral to the findings presented in this article in accordance with the policy described in the Instructions for Authors (https://academic.oup.com/plphys/pages/general-instructions) is: Andrew D.B. Leakey (leakey@illinois.edu).
