# Supplementary material for: Machine learning-enabled phenotyping for GWAS and TWAS of WUE traits in 869 field-grown sorghum accessions
Source: Plant Physiol. 2021 Jul 27;187(3):1481–500. doi: 10.1093/plphys/kiab346 (PMC9040483; doi:10.1093/plphys/kiab346)
Supplement: kiab346_Supplementary_Data [file kiab346_supplementary_data.zip › Ferguson et al Supplemental figures accepted.pdf]

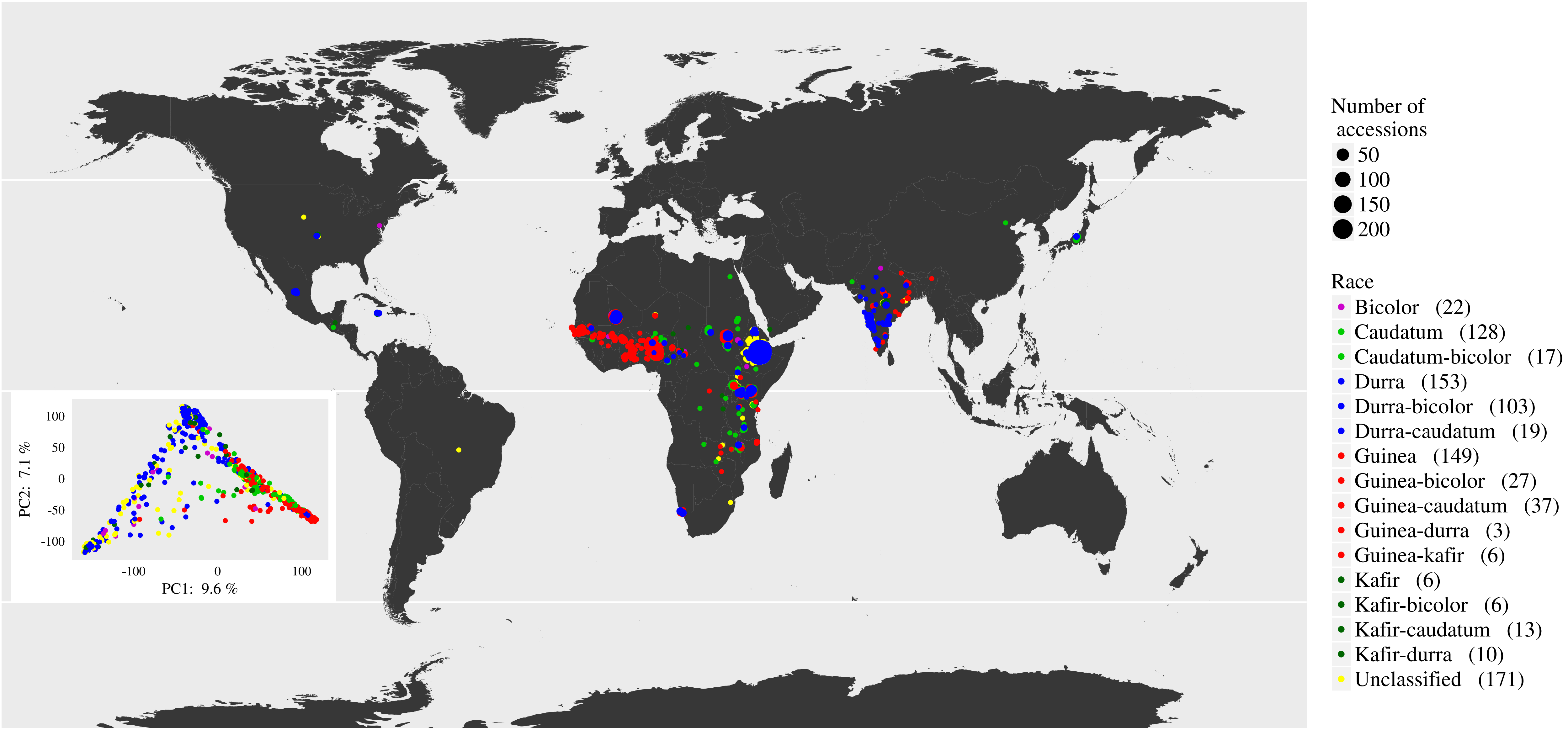

Supplemental Figure S1. Map showing the point of origin of all accession employed in this study.

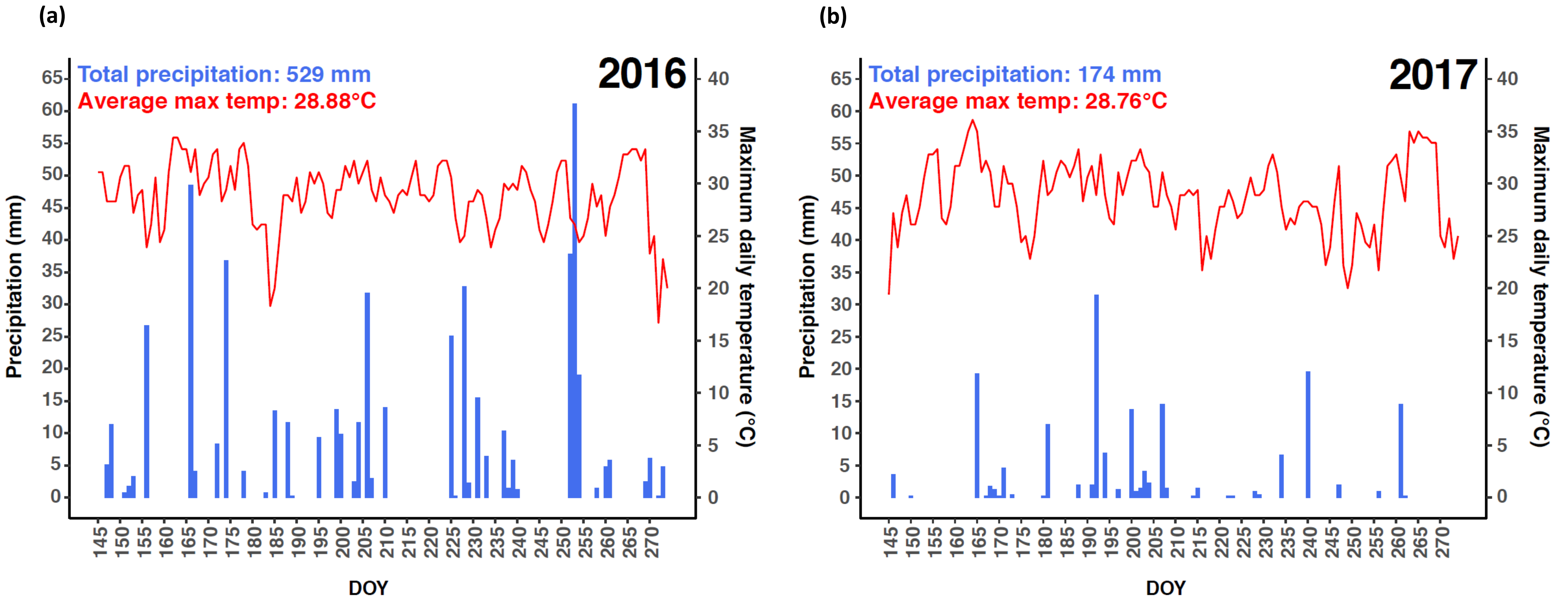

Supplemental Figure S2. Temperature and precipitation during growing seasons

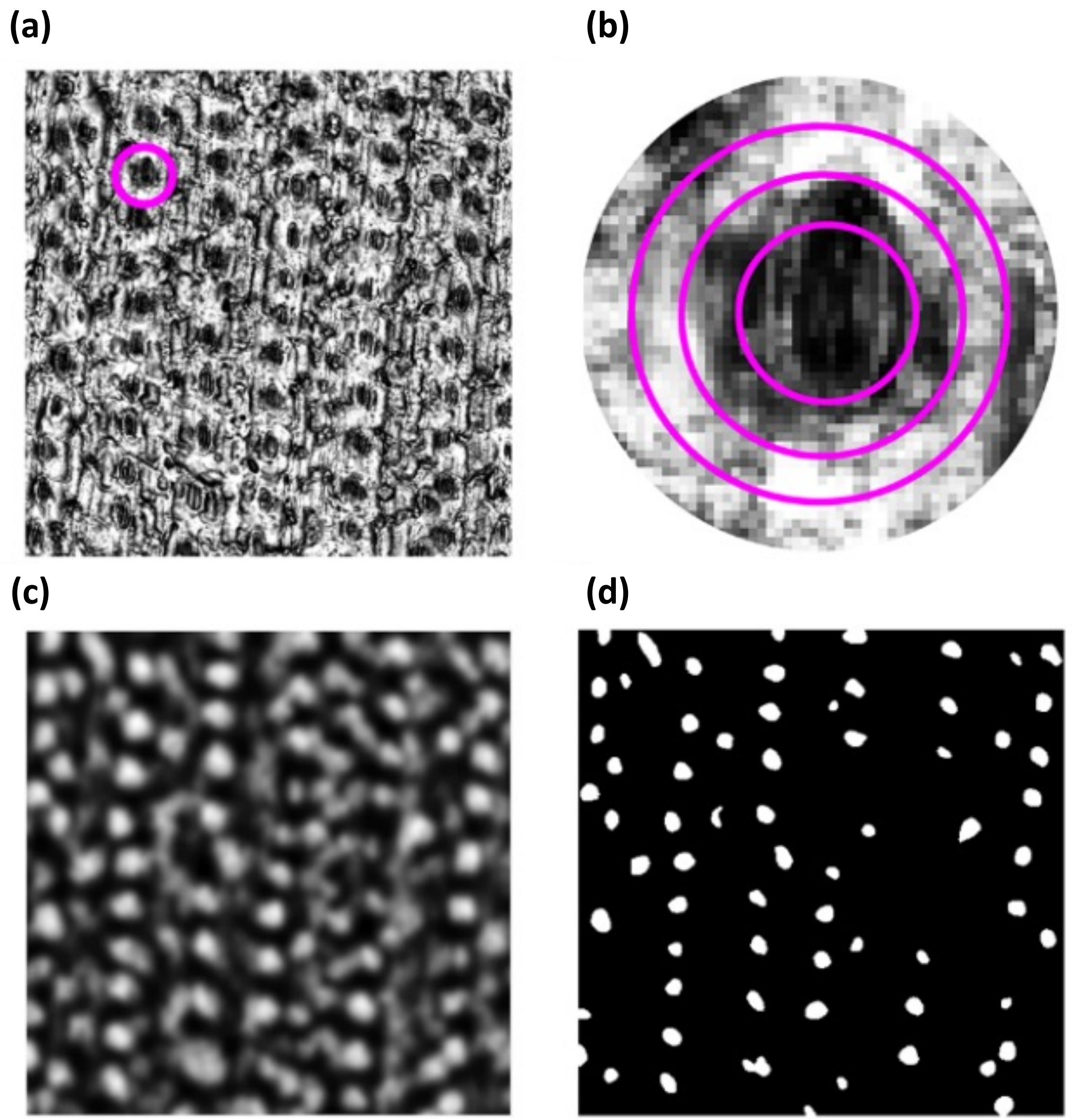

Supplemental Figure S3. Overview of stomatal counting machine learning method

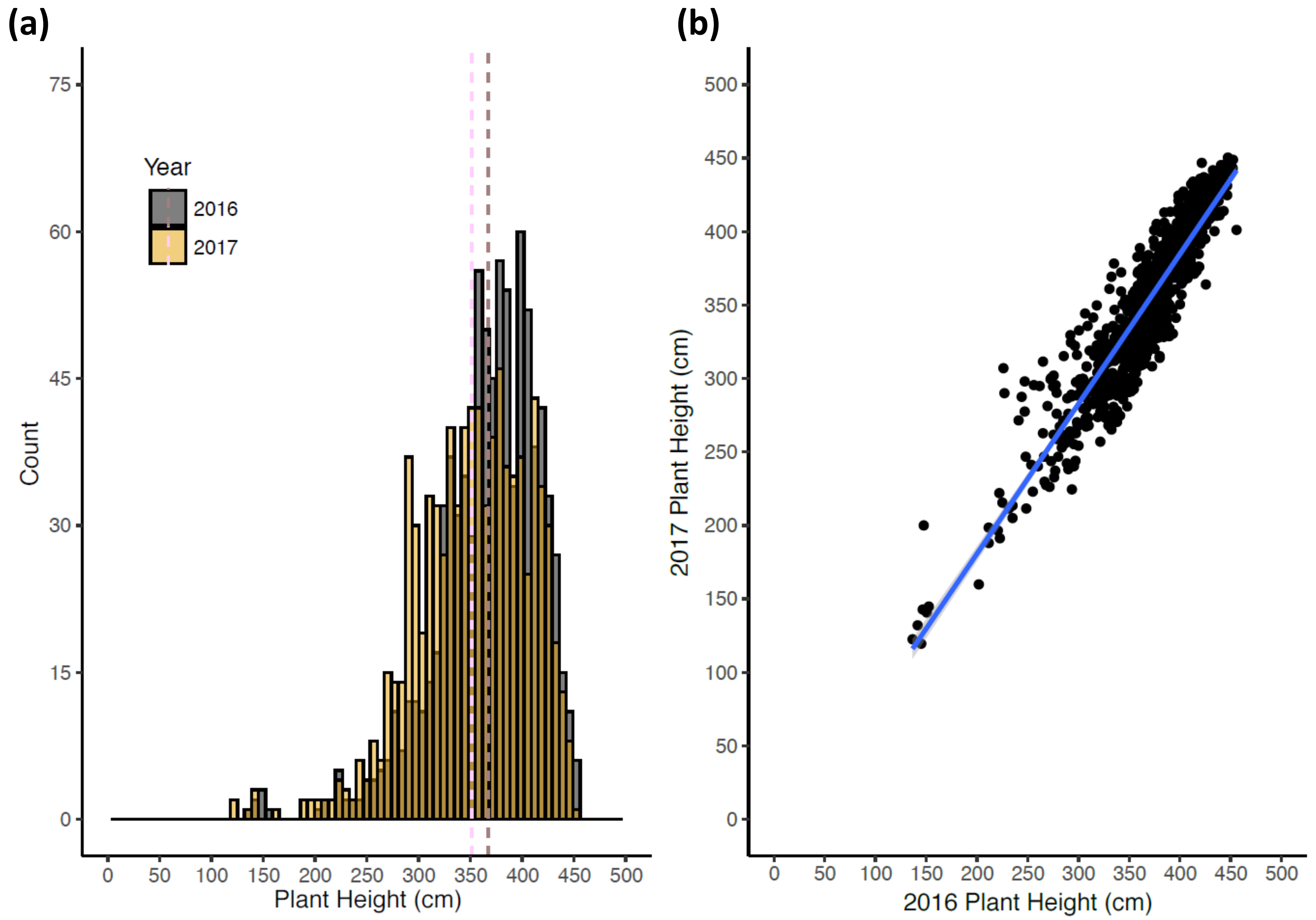

Supplemental Figure S4. Plant height correlation between growing seasons

SD 2017

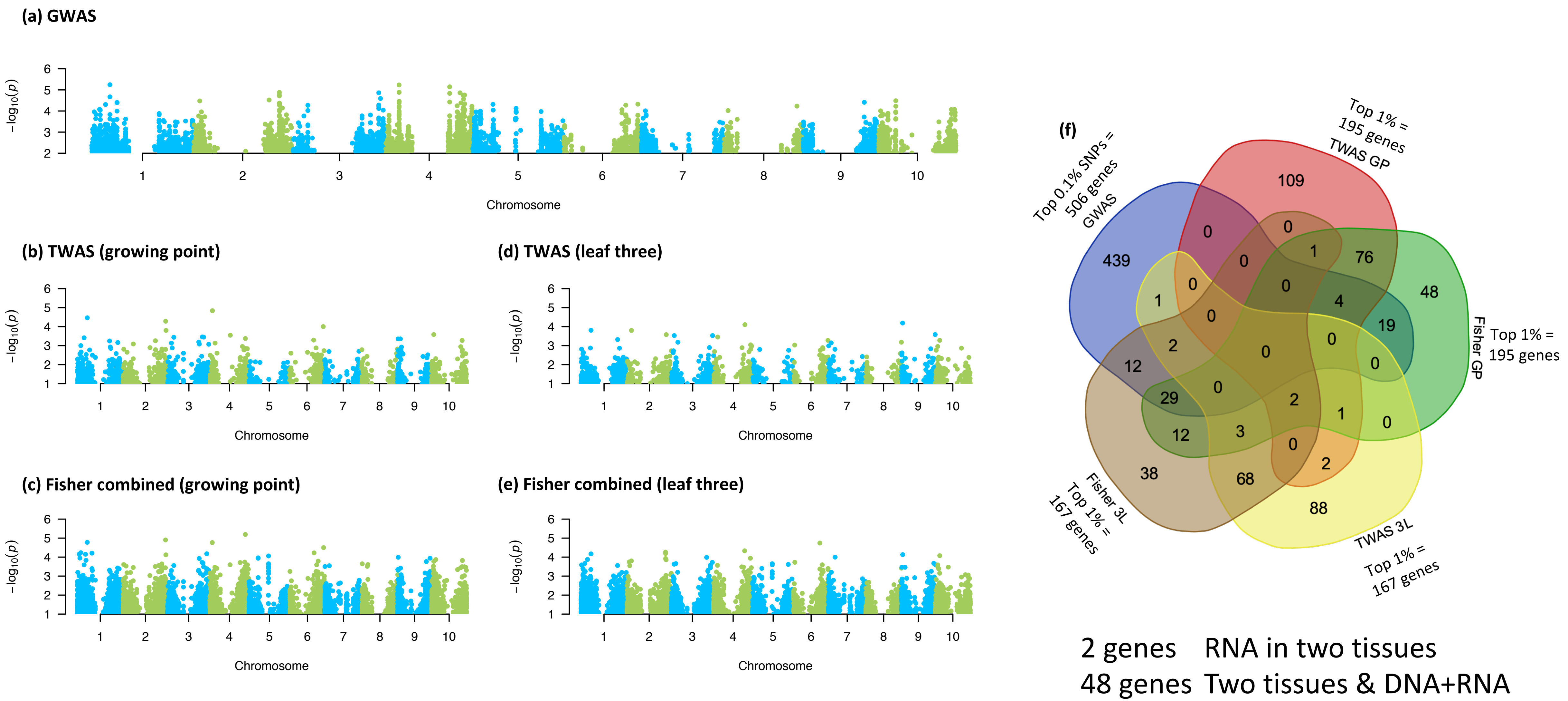

2 genes RNA in two tissues
48 genes Two tissues & DNA+RNA
7 genes DNA+RNA

Supplemental Figure S5. Mapping for stomatal density in 2017

SD Joint

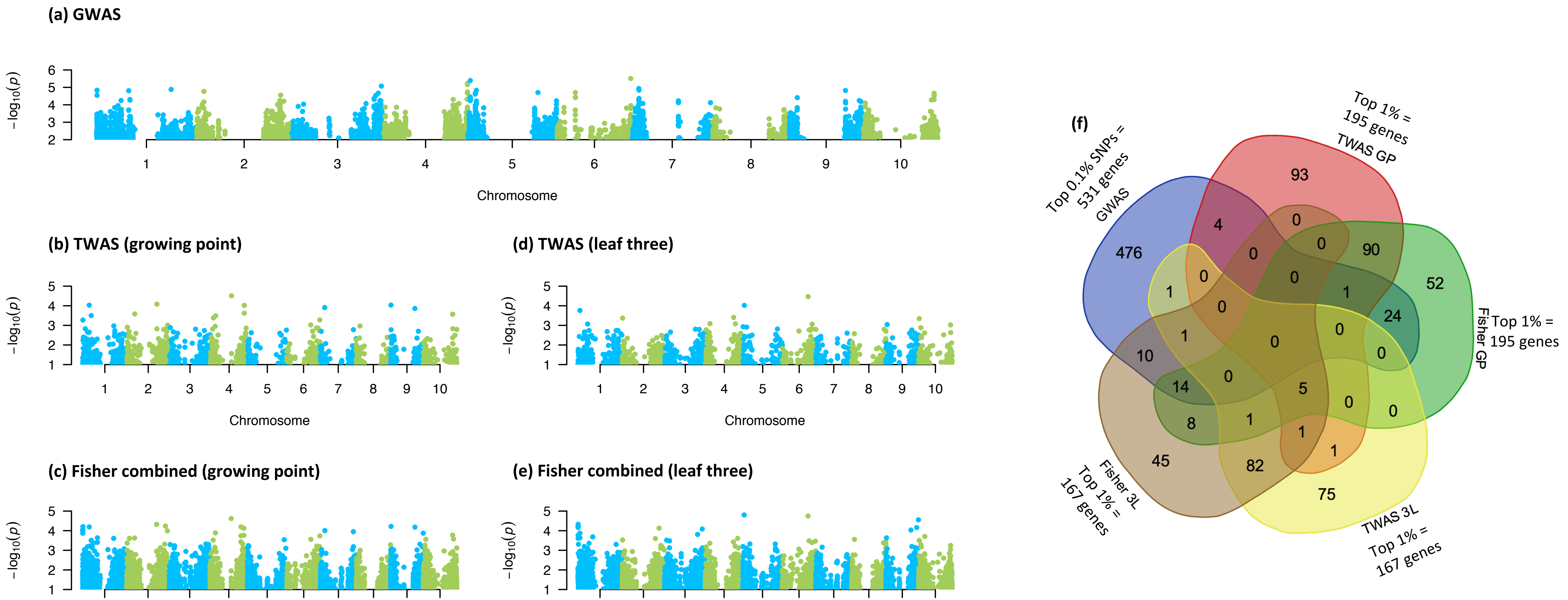

1 genes RNA in two tissues
29 genes Two tissues & DNA+RNA
7 genes DNA+RNA

Supplemental Figure S6. Mapping for stomatal density (joint year model)

SLA 2016

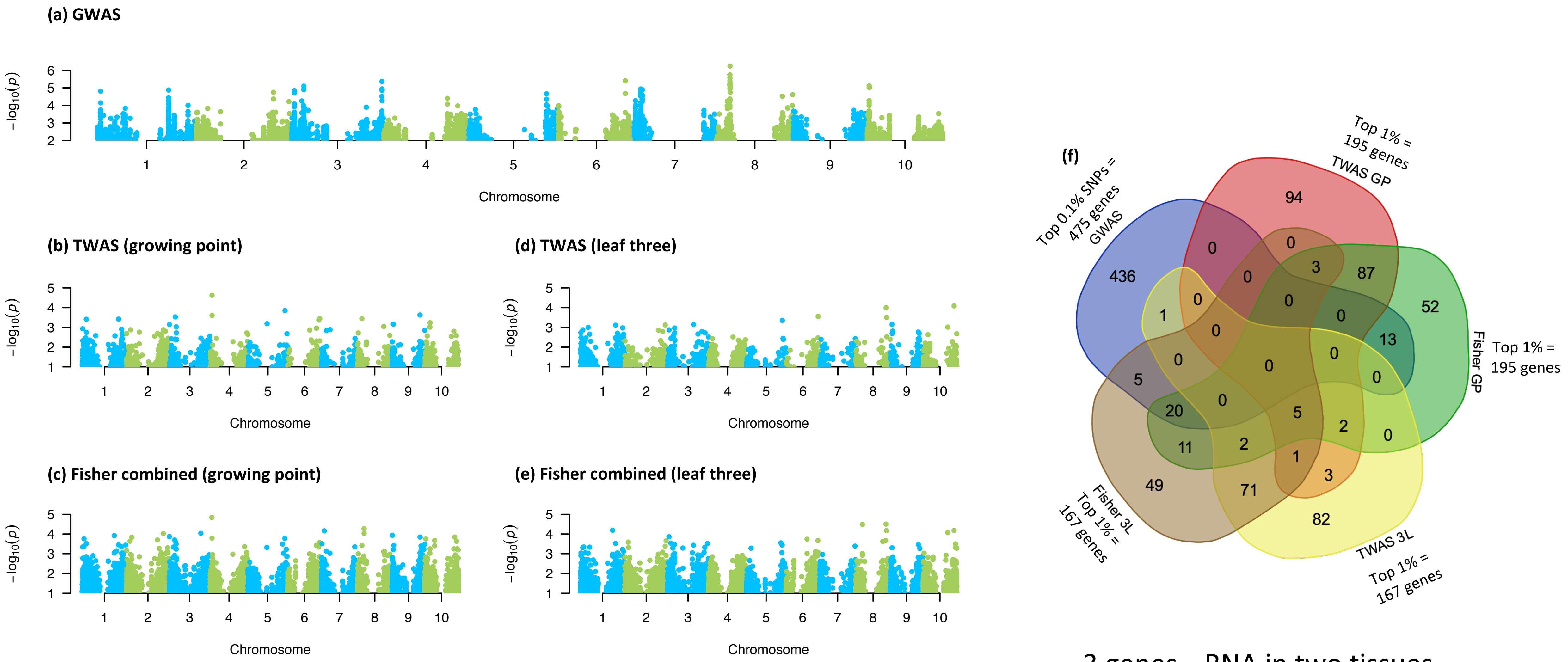

3 genes RNA in two tissues
44 genes Two tissues & DNA+RNA
1 genes DNA+RNA

Supplemental Figure S7. Mapping for specific leaf area in 2016

SLA 2017

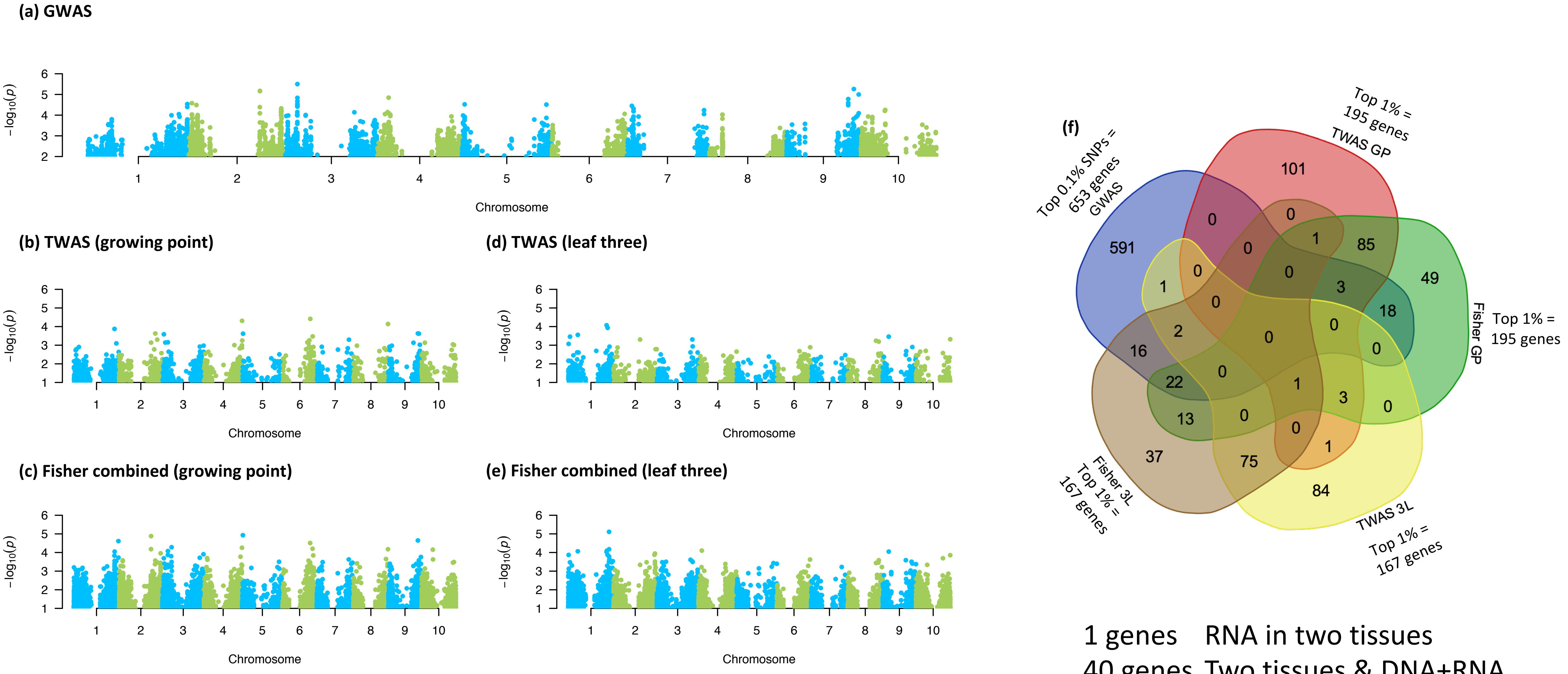

1 genes RNA in two tissues
40 genes Two tissues & DNA+RNA
6 genes DNA+RNA

Supplemental Figure S8. Mapping for specific leaf area in 2017

SLA Joint

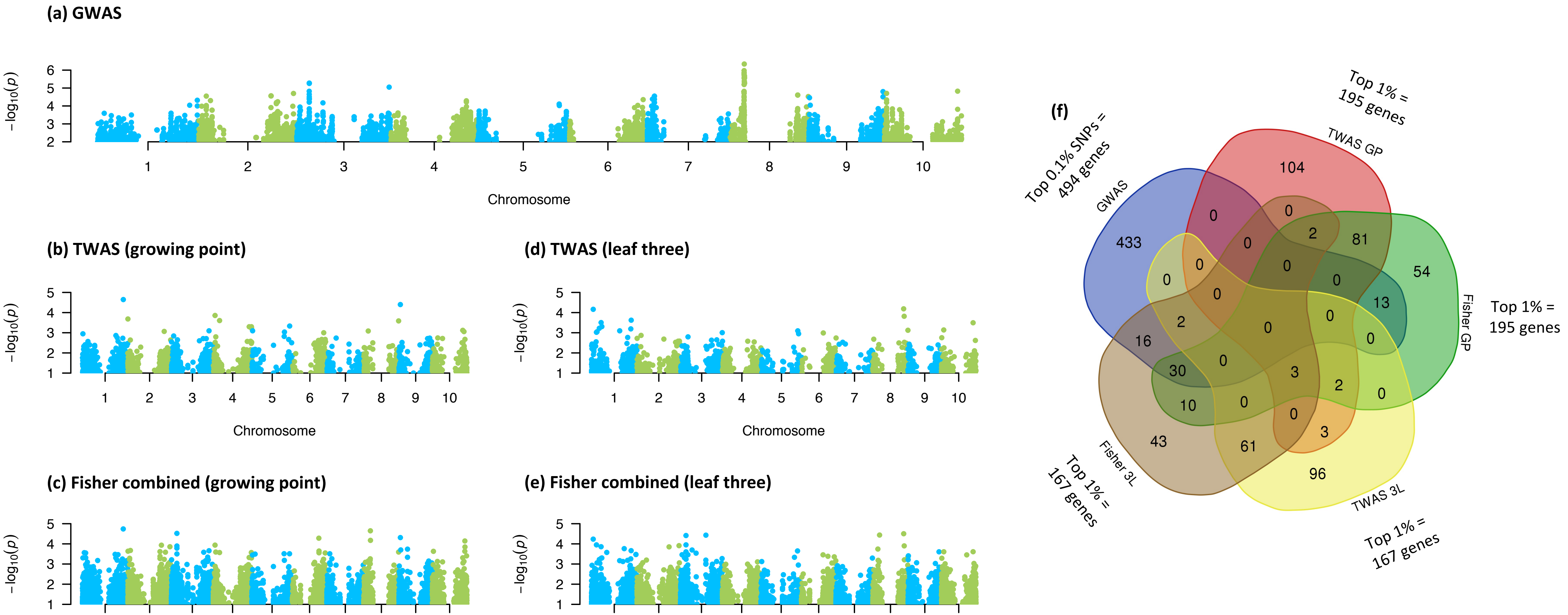

3 genes RNA in two tissues
47 genes Two tissues & DNA+RNA
2 genes DNA+RNA

Supplemental Figure S9. Mapping or specific leaf area (joint year model)

g_s 2017

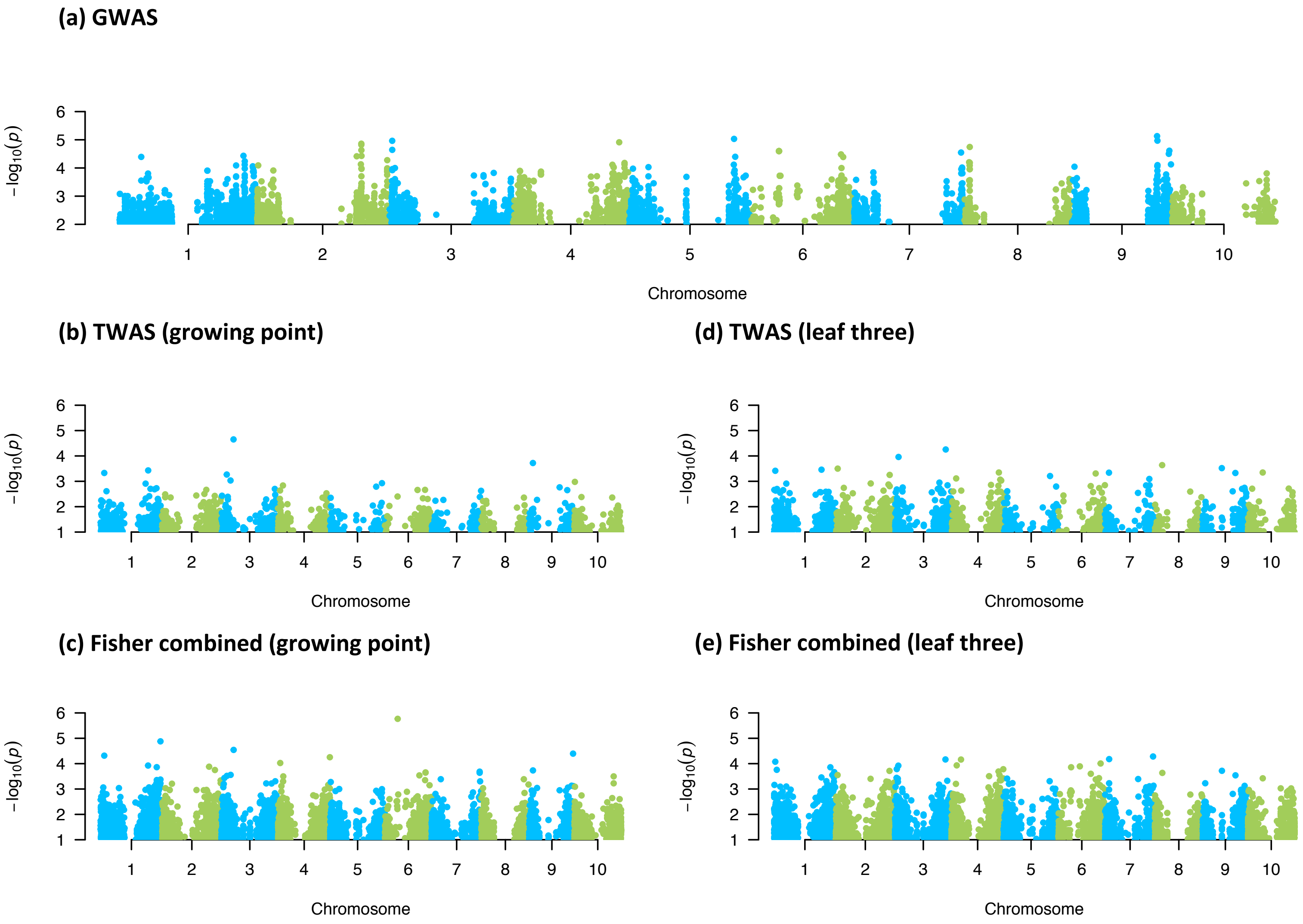

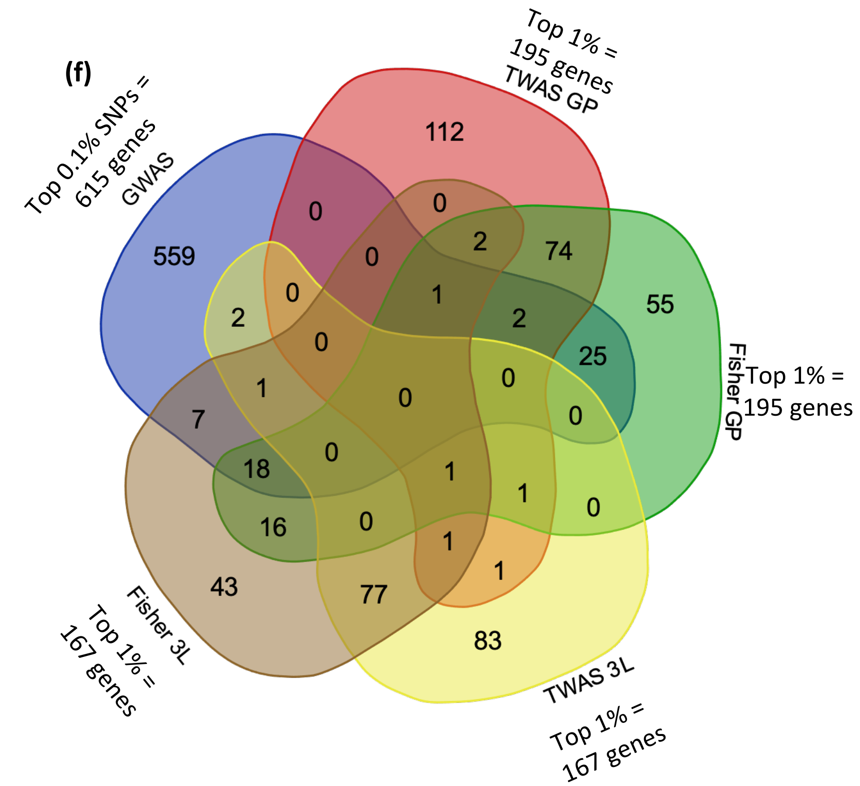

0 genes RNA in two tissues
39 genes Two tissues & DNA+RNA
4 genes DNA+RNA

Supplemental Figure S10. Mapping for stomatal conductance

A_N 2017

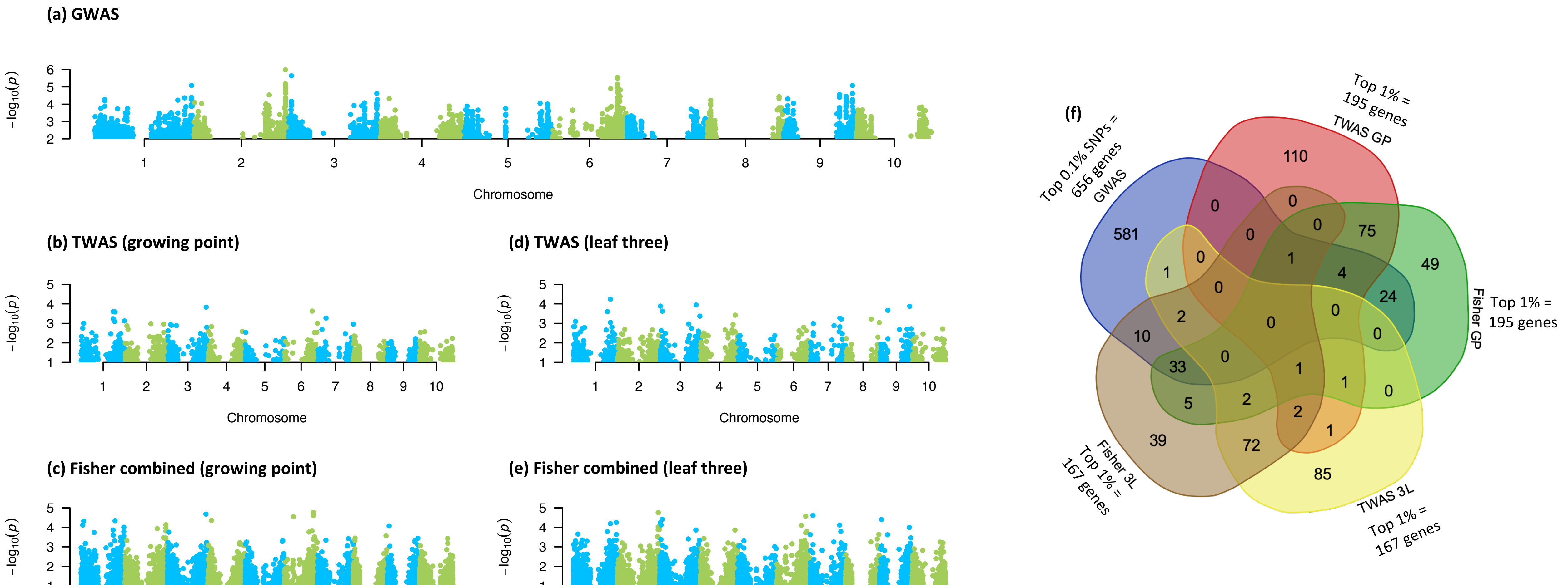

1 genes RNA in two tissues
45 genes Two tissues & DNA+RNA
7 genes DNA+RNA

Supplemental Figure S11. Mapping for photosynthesis

iWUE 2017

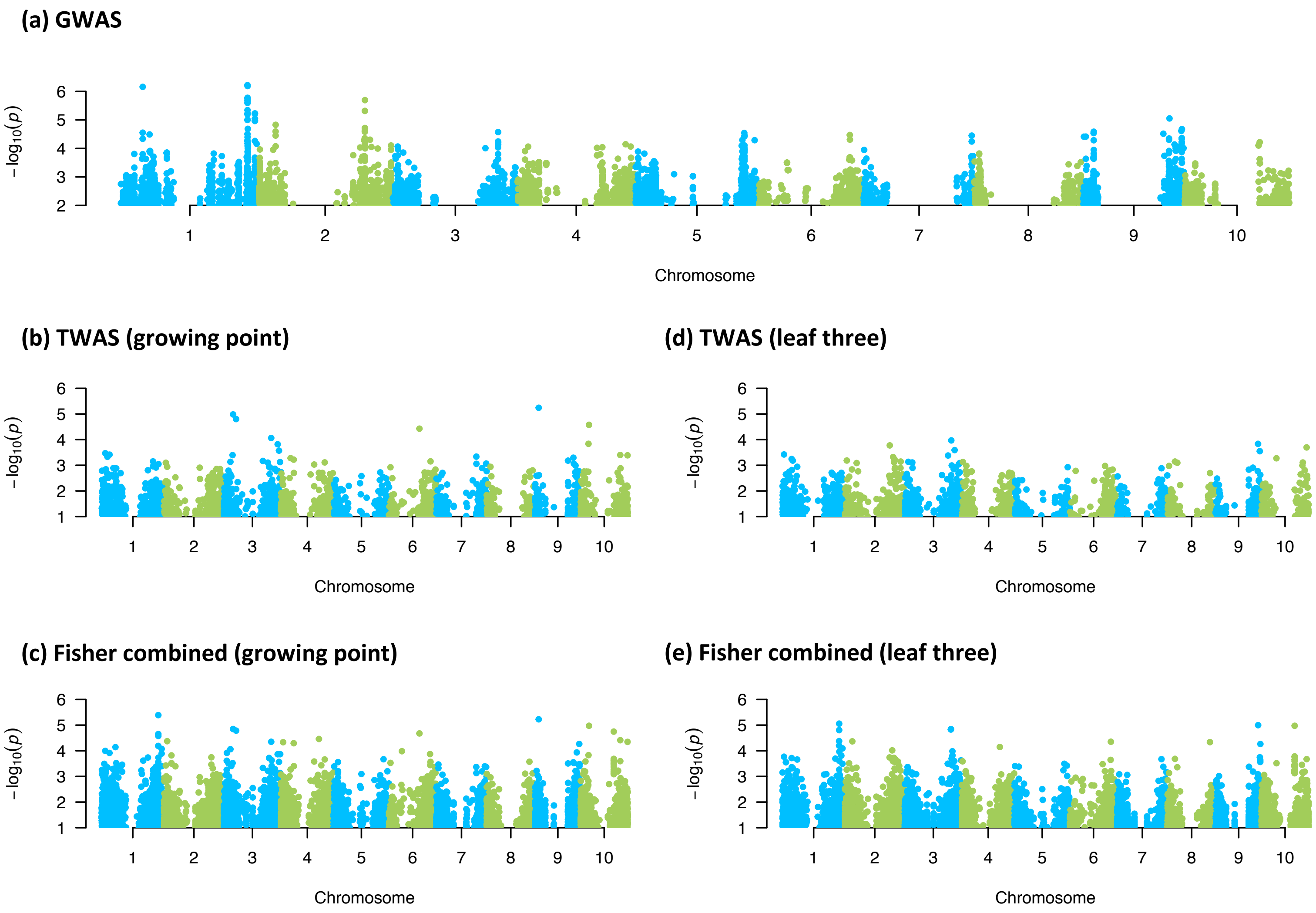

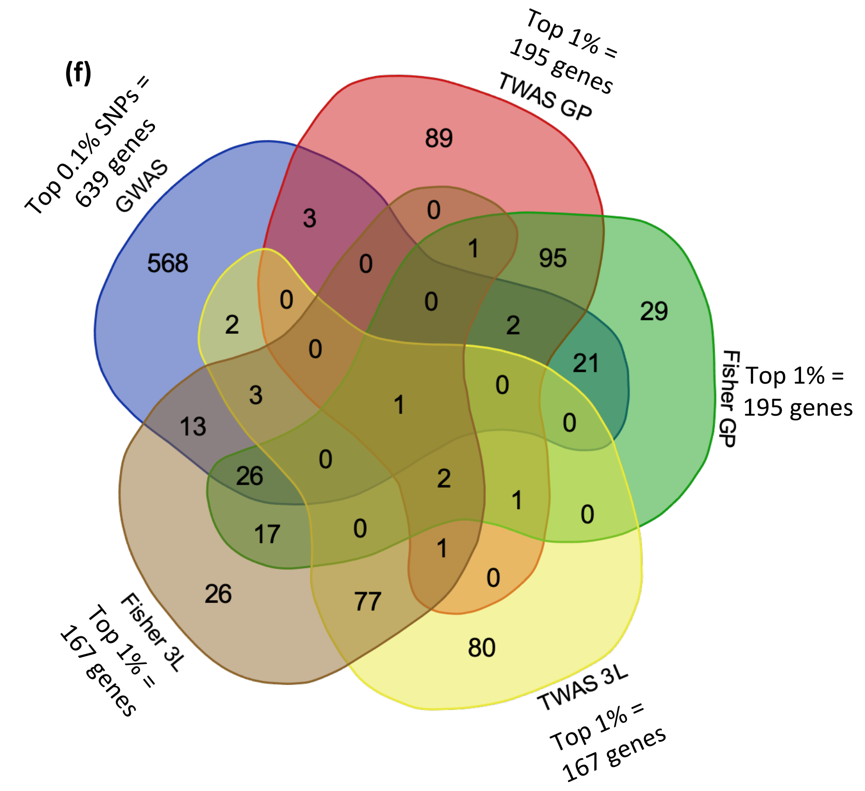

0 genes RNA in two tissues
49 genes Two tissues & DNA+RNA
10 genes DNA+RNA

Supplemental Figure S12. Mapping for intrinsic water use efficiency

c_i/c_a 2017

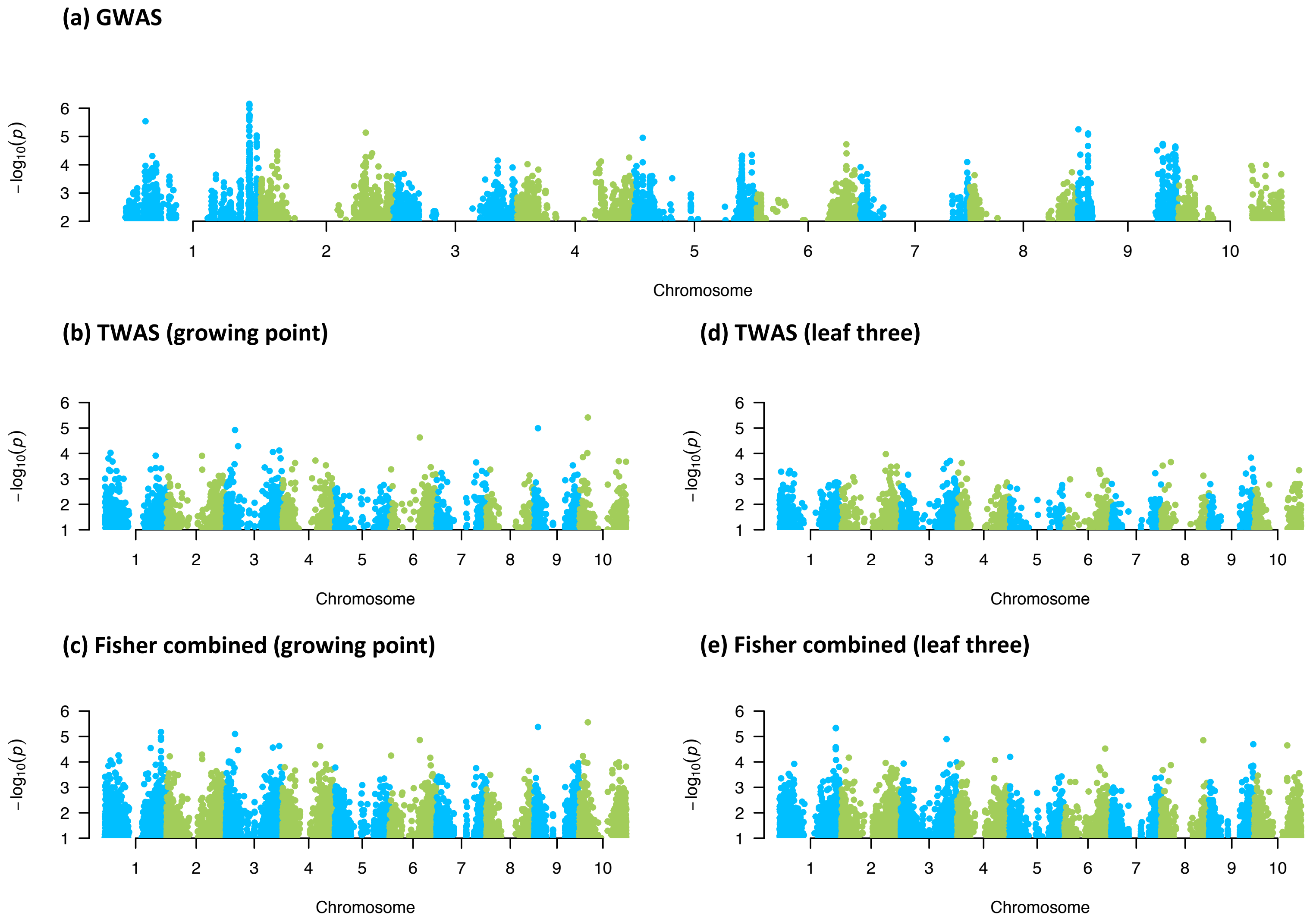

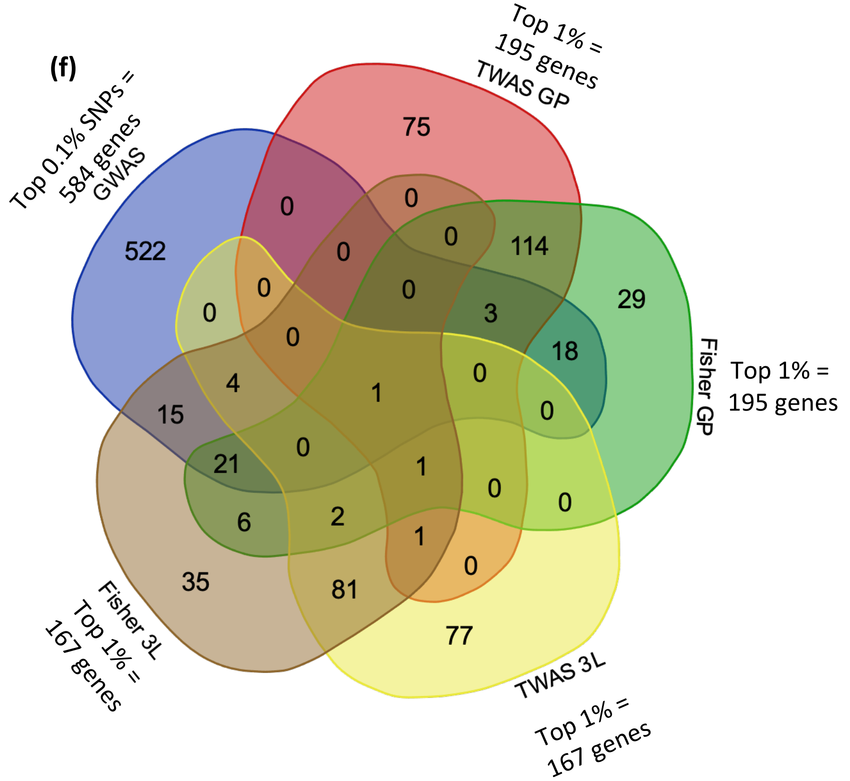

0 genes RNA in two tissues
32 genes Two tissues or DNA+RNA
7 genes DNA+RNA

Supplemental Figure S13. Mapping for the ratio of intracellular to ambient CO_2

(a)

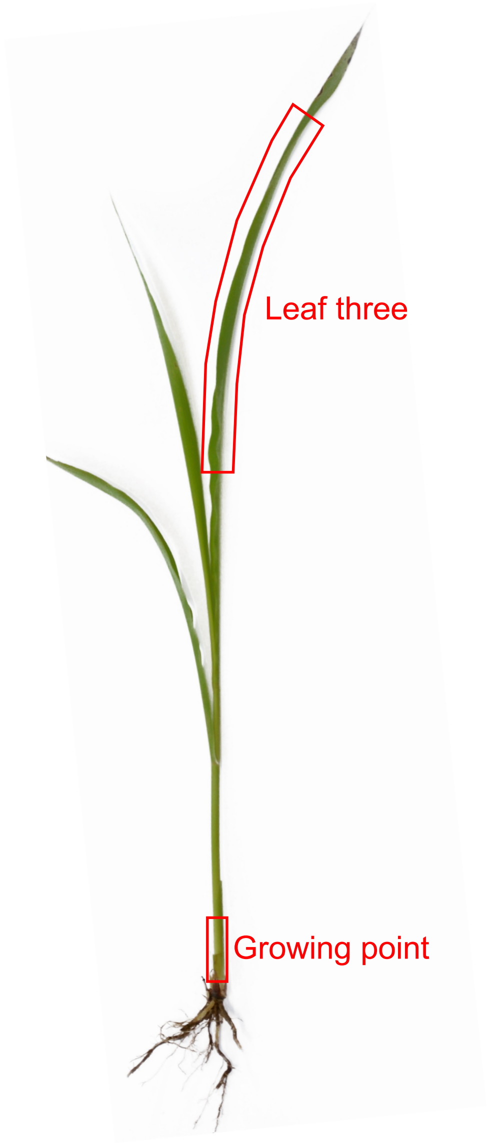

(b)

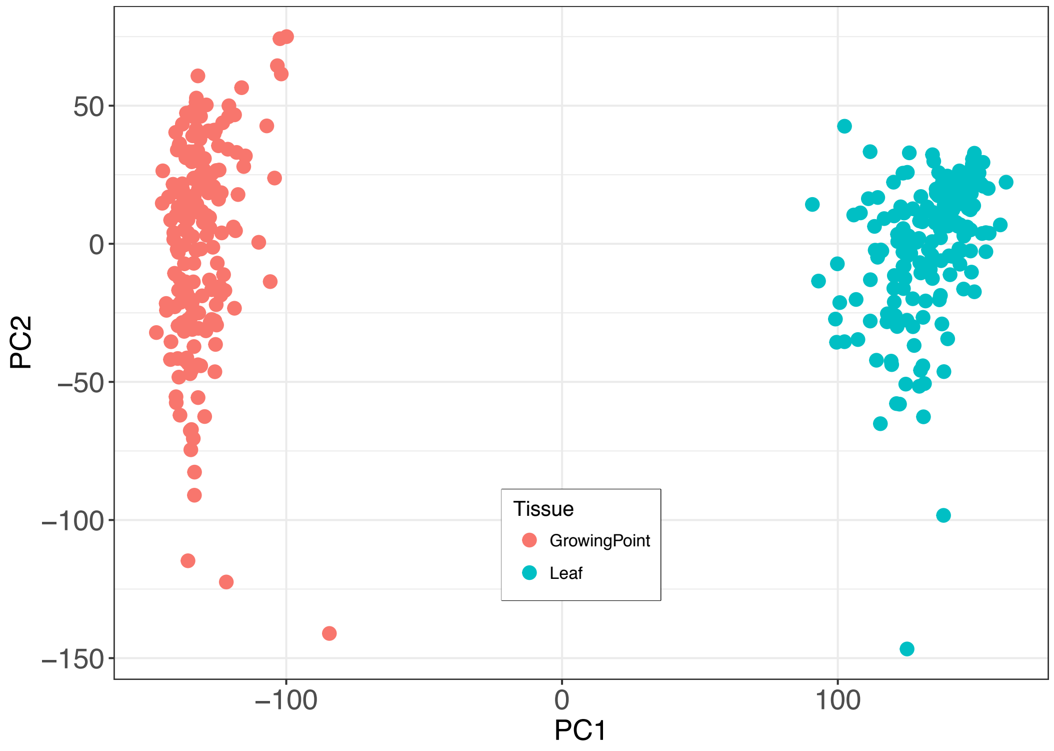

Supplemental Figure S14. Tissue harvested for RNAseq analyses

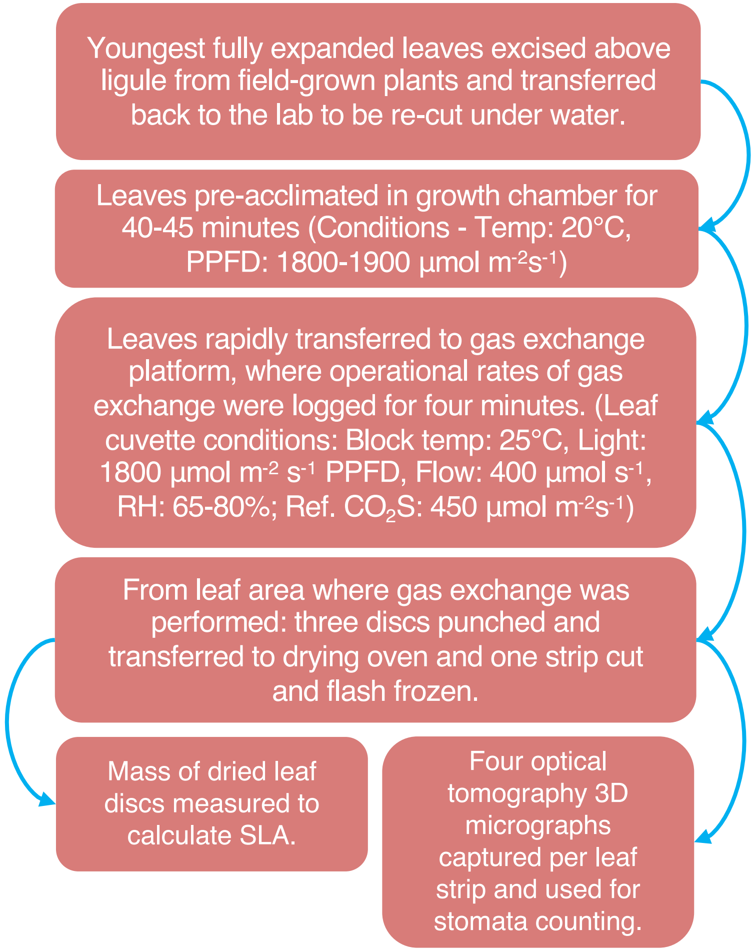

Supplemental Figure S15. Phenotyping pipeline

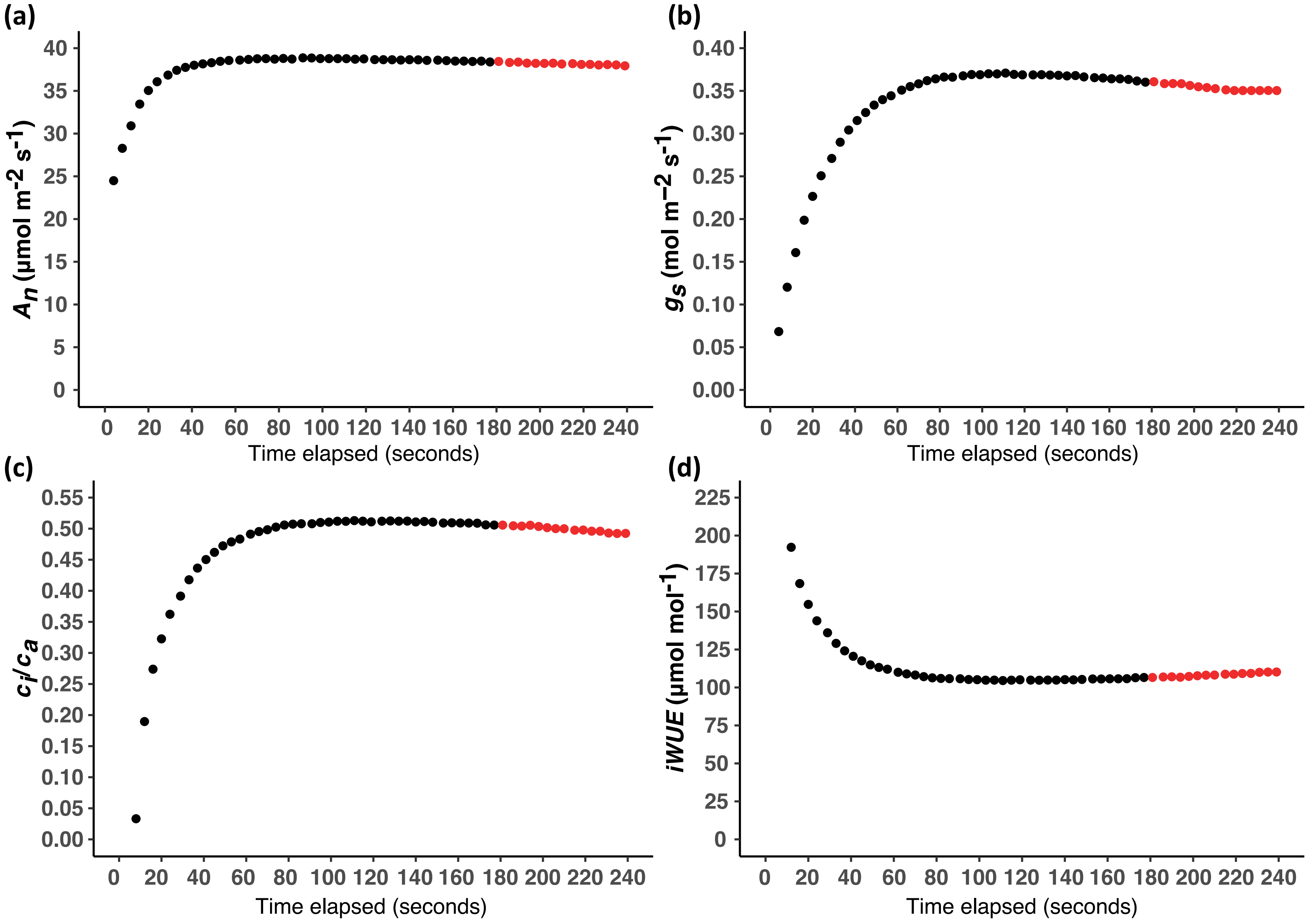

Supplemental Figure S16. Example of gas exchange data collection

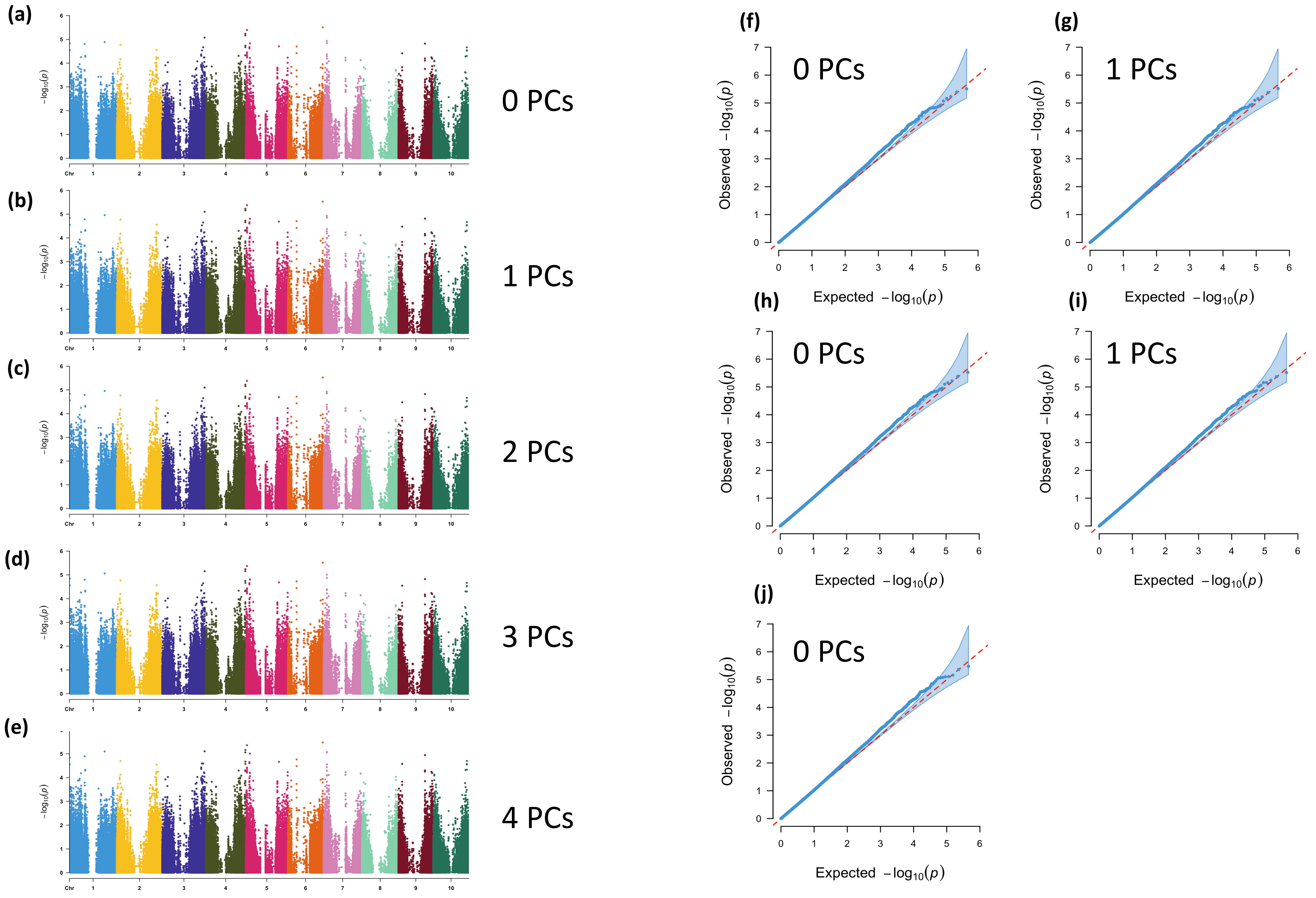

Supplemental Figure S17. Impact of inclusion of principle components for GWAS
